# Coping with climate change: limited behavioral responses to hot weather in a tropical carnivore

**DOI:** 10.1007/s00442-018-04329-1

**Published:** 2019-02-10

**Authors:** D. Rabaiotti, Rosie Woodroffe

**Affiliations:** 1 Institute of Zoology, Zoological Society of London, Regents Park, London, NW1 4RY UK; 2 Division of Biosciences, Department of Genetics, Evolution and Environment, Centre for Biodiversity and Environment Research, University College London, Gower Street, London, WC1H 0AG UK

**Keywords:** Activity, Behavior, Plasticity, *Lycaon pictus*, Temperature

## Abstract

Climate change is widely accepted to be one of the greatest threats to species globally. Identifying the species most at risk is, therefore, a conservation priority. Some species have the capacity to adapt to rising temperatures through changing their phenology, behavior, distribution, or physiology, and, therefore, may be more likely to persist under rising temperatures. Recent findings suggest that the African wild dog *Lycaon pictus* may be impacted by climate change, since reproductive success is consistently lower when pup-rearing coincides with periods of high ambient temperature. We used GPS collars, combined with generalized linear mixed-effects models, to assess wild dogs’ potential to adapt to high ambient temperatures through flexible timing of hunting behavior. On days with higher maximum temperatures, wild dogs showed lower daytime activity and greater nocturnal activity, although nocturnal activity did not fully balance the decrease in daytime activity, particularly during the denning period. Increases in nocturnal activity were confined mainly to moonlit nights, and were seldom observed when packs were raising pups. Our findings suggest that nocturnal activity helps this cursorial hunter to cope with high daytime temperatures. However, wild dogs appear not to use this coping strategy when they are raising pups, suggesting that their resource needs may not be fulfilled during the pup-rearing period. Given that moonlight availability—which will not change as the climate changes—constrains wild dogs’ nocturnal activity, the species may have insufficient behavioral plasticity to mitigate increasing diurnal temperatures. These findings raise concerns about climate change impacts on this endangered species, and highlight the need for behavior to be considered when assessing species’ vulnerability to climate change.

**Electronic supplementary material:**

The online version of this article (10.1007/s00442-018-04329-1) contains supplementary material, which is available to authorized users.

## Introduction

As the climate warms, declines in wildlife population sizes and species richness, coupled with shifts in species distribution, are predicted to occur worldwide, across a wide variety of flora and fauna (Walther et al. 2002; Bellard et al. 2012). A number of studies have shown that climate change is already having an impact across a range of species, including phenological shifts (Parmesan and Yohe 2003), geographic range shifts and contractions (Parmesan 2006; Tingley et al. 2012), and population and species extinctions (Parmesan 1996; Beever et al. 2003; Pounds et al. 2006, Sinervo et al. 2010).

Behavioral plasticity has the potential to buffer climate change impacts on wildlife. Species as diverse as the koala *Phascolarctos cinereus* (Briscoe et al. 2014), Arabian oryx *Oryx leucoryx* (Hetem et al. 2012), and Southern fiscal *Lanius collaris* (Cunningham et al. 2015) respond to high ambient temperatures by changing their behaviors. Such plasticity has the potential to mitigate climate change impacts (Martin et al. 2015). However, these thermoregulatory behaviors often come with a fitness cost, such as a decrease in foraging time, or reduced vigilance, which may impact survival and reproduction as the climate warms (Sinervo et al. 2010; du Plessis et al. 2012; Cunningham et al. 2013, 2015; Turbill and Prior 2016). Species’ behavioral responses are, therefore, likely to be key in determining the extent to which a species is impacted by rising temperatures.

Species can adapt to climate change in three ways: adaptation in time, whereby they shift their phenology, moving the timing of critical life events, such as breeding, in response to changing seasonality; adaptation in space, where species’ ranges shift to remain within appropriate climatic conditions; and adaptation in self, whereby individuals of a species alter their behavior or physiology in response to changing temperatures (Foden et al. 2013, Pacifici et al. 2015). Such adaptation to climate change can either occur through evolutionary change, whereby traits are inherited through generations, or through phenotypic plasticity, whereby species’ traits are altered without altering their genes, often within the lifetime of an individual, in ways which mitigate climate change impacts. These traits can include species’ physical characteristics, geographic range, physiology or behaviors, as well as changes to species phenology. However, most studies and models of climate change vulnerability either look solely at the correlation between species occurrence and climatic variables, ignoring the underlying mechanisms of climate change impacts (Kearney et al. 2010; Pacifici et al. 2015), or else focus on physiological rather than behavioral traits (Bellard et al. 2012). In studies where models of climate responses have incorporated behavioral thermoregulation, they have generally been focused on ectotherms (Kearney et al. 2009; Huey et al. 2012).

Ambient temperatures are predicted to rise significantly over the next 50 years, and Africa is projected to experience greater warming than the global average (IPCC 2014). African species may, therefore, face particularly high extinction risks under climate change. We, therefore, explored the role of behavioral plasticity in a species vulnerable to climate change, the African wild dog *Lycaon pictus*.

The African wild dog is a highly social carnivore, with pack members cooperating to hunt, raise young, and defend resources (Creel and Creel 2002). The species is globally endangered; the wild population numbers fewer than 700 packs, confined to just 7% of the species’ former range within sub-Saharan Africa (Woodroffe and Sillero-Zubiri 2012). Being highly mobile, with a relatively flexible diet, the species has few of the traits typically associated with climate change vulnerability (Bellard et al. 2012; Pacifici et al. 2015). Indeed, it has been suggested that wild dogs may benefit from rising temperatures that may reduce the ability of large-bodied prey, for example, wildebeest (*Connochaetes taurinus*), to outrun smaller-bodied predators such as African wild dogs (Creel et al. 2016). However, demographic evidence indicates consistently harmful effects of hot weather on wild dogs, with high ambient temperatures associated with lower recruitment across multiple populations (Woodroffe et al. 2017). Wild dogs hunt less on hot days, and these demographic impacts may reflect consequently lower food intake (Woodroffe et al. 2017).

A primary opportunity for African wild dogs to adapt to rising temperatures is through a change in self. Shifting activity to night-time, which is cooler than daytime, has been suggested as one of the primary ways large mammals may mitigate the impacts of rising temperatures (Fuller et al. 2016). Wild dogs are crepuscular, hunting for 1–2 h at dawn and dusk (Creel and Creel 2002; Cozzi et al. 2012; Woodroffe et al. 2017). However, nocturnal hunts also occur (Cozzi et al. 2012), especially on moonlit nights, and increasing the frequency of nocturnal hunting might allow wild dogs to exploit lower night-time temperatures, potentially offsetting climate change impacts. Such a coping strategy might be especially important during the 3-month period each year when pups are confined to a den and adults’ energy demands are highest (Woodroffe et al. 2017).

We explored the potential for African wild dogs to cope with high daytime temperatures by increasing nocturnality. We predicted that wild dogs’ night-time activity and ranging distance would be greater (i) following hot days, (ii) on moonlit nights, and (iii) during the pup rearing (denning) period.

## Materials and methods

### Study area

The study area covers Laikipia county in Northern Kenya (37°2′E, 0°6′N), and parts of neighboring Samburu, Isiolo and Baringo counties. The habitat mainly comprises semi-arid bushland and savannah, with livestock farming and tourism as the primary land uses. Despite not being formally protected, as the majority of land is under private or community ownership, many landowners promote wildlife and tourism alongside pastoralism and ranching activities, leading to high levels of wildlife abundance and diversity. Mean annual rainfall is 590 mm varying from around 400 mm per year in the North East to over 900 mm per year in the South West, with highly variable seasonality. Daily maximum temperatures range from 25 to 36 °C with minimum temperatures falling between 12° and 17 °C (Caylor et al. 2016). Wild dogs in this population feed primarily on dik-diks (*Madoqua spp.*) and impala (*Aepyceros melampus*; Woodroffe et al. 2007).

### Field data collection

Data were collected between 2011 and 2016. We fitted GPS-collars (GPS-plus, Vectronic Aerospace GmbH, Berlin, Germany) to 15 wild dogs in 8 different packs; there was only one active GPS collar on each pack at any one time. Wild dogs were darted from a vehicle from a distance of 10–15 m—further details of collar deployment methods are provided in Woodroffe (2011). Individual wild dogs were GPS-collared for an average of 207 days (SD = 126); details of the monitoring dates and packs of individual study animals are shown in Online Resource 1. The collars contained accelerometers that recorded average acceleration in two (unspecified) dimensions every 5 min on a scale of 0–255. GPS collars were programmed to record locations at specific times throughout the day and night. The programmed number of GPS locations per 24-h period varied between individuals from 6 to 13 (Online Resource 2). The GPS collars incorporated VHF beacons, and additional pack-members were also fitted with VHF collars to help in locating the pack in case of GPS collar failure. We downloaded the data from GPS collars over a remote VHF link. We also made visual observations of wild dog behavior throughout the study which corroborated the timing of periods of activity recorded by the collars. We identified denning dates, and dates when packs moved den sites, using GPS collar data, based on the distinctive movement pattern of repeatedly returning to the same location (Woodroffe 2010). African wild dog packs switch den sites multiple times throughout a denning period—with an average of 5 den moves (SD 2.85) from birth until the last den is abandoned when pups are approximately 3 months old (Woodroffe et al. 2017). Because den site changes repeatedly during the denning period, the number of days spent at each den site is not strongly correlated with pup age (Online Resource 3).

### Variables analysed

We analyzed two types of dependent variable: activity, and distance traveled. We calculated activity by summing accelerometer data for each 5-min period from the two planes and then converting into percentages of the maximum value (510) to give a measure of activity from 0 to 100. Mean daytime activity was then calculated for the period between sunrise and sunset at the study site, and mean night-time activity was calculated for the period between sunset and sunrise (obtained from the US Naval Observatory (http://www.usno.navy.mil/). Mean activity across each 24-h period between successive sunrises was also calculated.

We calculated distance traveled using GPS-collar locations. To avoid the influence of differing numbers of GPS points (i.e., spatiotemporal resolution) on the measures of distance traveled, the same six time points were used across all individuals: 06:30, 08:00,13:00, 18:00, 19:30, and 01:00 (see Online Resource 2). We estimated distance traveled by calculating the distance between consecutive GPS locations, then summing the distances 06:30–08:00–13:00–18:00 to give daytime distance traveled, 18:00–19:30–01:00–06:30 to give night-time distance traveled, and 06:30–08:00–13:00–18:00–19:30–01:00–06:30 to give 24-h distance traveled (Online Resource 2). Periods when a GPS collar failed to record a location at one or more of the selected time points were discarded; the probability of at least one missing location was greater for longer periods; hence, more 24-h periods were discarded than daytime or nightime periods, leading to a lower sample size of 24-h periods (Online Resource 3).

Typically, one pack member stays at the den guarding the pups while the rest of the pack hunts (Malcolm and Marten 1982; Creel and Creel 2002). To better represent the hunting behavior of the pack during denning periods, we excluded days when collared animals remained < 200 m from the den at times when the pack would normally hunt (06:30–08:00 or 18:00–19:00). Online Resource 3 shows the numbers of observations of activity and distance traveled analyzed for each individual.

We tested the hypothesis that wild dogs nocturnal activity and ranging distance were greater following hot days by comparing activity and distances traveled with dry bulb daily maximum air temperature (the highest temperature (in  °C) within a 24-h period). In models of nocturnal activity and distance traveled, maximum daily temperature referred to the preceding period of daylight. As heat stress is also effected by capacity for evaporative cooling, we also included total daily rainfall (mm) as an independent variable. Temperature and rainfall were measured at Mpala Research Centre (37°2′E, 0°6′N), within the study area (Caylor et al. 2016). We tested the hypothesis that wild dogs were more active and traveled further on moonlit nights by comparing activity and distances traveled with levels of moonlight, which were estimated (from data at http://www.usno.navy.mil/) as the number of hours the moon was in the sky between sunset and sunrise (0–12 h), multiplied by the proportion of the full moon that was illuminated (0–1) to give a combined moonlight variable measured in full-moon hours (0–12; Online Resource 5). For example, a moonlight value of 12 would indicate a full moon for 12 h between sunset and sunrise. The moonlight variable did not account for cloud cover as these data were not available; however, we would expect cloud cover to be correlated with rainfall. We tested the hypothesis that wild dogs were more active and traveled further during the denning period by comparing activity and distances traveled with reproductive status, was represented as denning/not denning. As behavior during he denning period is impacted by days since occupying a particular den site and pup age (in days) (Woodroffe et al. 2017), these were included as independent variables in models of the denning period (Online Resource 4).

### Statistical analyses

We used generalized linear mixed-effects models to investigate the associations between independent variables (maximum daily temperature, daily rainfall, moonlight, pack reproductive status, days spent at a den site, and pup age) and dependent variables (activity, distance traveled). In addition to these fixed effects, we also included individual identity as a random effect. One set of models considered both denning and not denning periods, and included daily rainfall, maximum daily temperature, moonlight, and denning (yes/no) as independent variables. A second set of models considered data only from the denning periods. These denning-specific models included daily rainfall, maximum daily temperature, moonlight, pup age, and the number of days since occupying the den site (Online Resource 4). We also examined two-way interactions where they were considered to be potentially ecologically relevant (Online Resource 4).

Due to the distribution of the model residuals (Online Resource 6), gamma generalized linear mixed-effects models were fitted to all datasets. Residuals were checked for normality and heterogeneity by eye using Q–Q plots (Online Resource 7). An information theoretic approach was used to select the top model set, comparing the corrected AIC (AICc) between models. As this approach yields several acceptable models, a model averaging approach was used to determine final estimates (Burnham and Anderson 2002). The relative importance of fixed effects was evaluated by averaging parameter estimates across the top models (ΔAIC ≤ 5). As some literature suggests that ΔAIC ≤ 2 (Harrison et al. 2018) should be used as a cut off, we also report the number of models in the ΔAIC ≤ 2 set that each fixed effect was included in. Tables of the top models are presented in Online Resource 8. All independent variables were tested for intercorrelation and all Pearson’s correlations were found to be below *r* = 0.25 (Online Resource 9). We carried out all analyses in R version 3.3.2 (R Core Team 2018) and used the *lme4* (Bates et al. 2015) package to fit models. The package *MuMIn* (Barton 2002) was used for model averaging and model selection.

### Projections

To examine how wild dog activity and distance traveled might change in the future under climate change we projected the models into the year 2070 under a variety of climate scenarios. We first defined the study area by drawing minimum convex polygons around the GPS locations used to calculate the distance traveled data for each wild dog, and these minimum convex polygons were then merged to give a single (non-convex) polygon. Current estimates (representative of the years 1960–1990) and future projections (for the year 2070) of mean monthly maximum temperature (the monthly mean of daily maximum temperature) and total monthly rainfall for the study site, at a spatial resolution of 30 arc seconds, were taken from WorldClim 1.4 (Hijmans et al. 2005). For future projections, the dataset from the HADGEM2 climate model was used for both the best case scenario (Representative Concentration Pathway 2.6) and worst case scenario (Representative Concentration Pathway 8.5) (IPCC 2014) predictions. For both WorldClim estimates (the current estimates and the future projections), we calculated mean daily maximum temperature and mean daily rainfall by averaging the estimates for all 12 months of the year for each pixel and dividing total monthly rainfall by 30 to give a daily average. A diagram illustrating these calculations can be found in Online Resource 10.

Estimated current mean daily maximum temperature for the study site from WorldClim estimates was significantly lower (mean 2.7 °C lower) than the average daily maximum temperature taken from the weather station throughout the duration of this study (2011–2016), and estimated mean daily rainfall was higher (mean 0.4 mm) than the average value from the weather station throughout the duration of this study (2011–2016). These differences are likely due to the interpolation method used by WorldClim, based on poor weather station coverage across Africa (Bogale et al. 2011), as well as the fact that WorldClim estimates were calculated for the whole study site rather than the location of the weather station alone. As the WorldClim current estimates differed consistently from the measurements from the weather station in the study site, which were used to build our models, two predicted change variables were created by subtracting the WorldClim estimate of current temperature across the study site, at a resolution of 30°, from the projected future temperature across the study site, at a resolution of 30°. This procedure was then repeated for current and future rainfall. The average projected changes in temperature and precipitation were calculated by taking the mean across all pixels across the study site in the predicted change rasters. We then calculated the average activity and distances traveled under current temperature conditions using the mean values from the weather station on site, and calculated the values predicted by the model of denning and non-denning periods combined, and for the denning period only. To get future predictions of activity and distances traveled, we added the predicted change in temperature and precipitation, derived from the WorldClim estimates, to average temperature and precipitation from the weather station data, and used these values to calculate activity and distances traveled predicted by the models of denning and non-denning periods combined, and the non-denning period only, under the expected changes in temperature and precipitation. Current mean activity and distances traveled was then subtracted from future activity and distances traveled to get the predicted future change in the dependant variables between now and 2070. Analyses were carried out using the *rgdal* (Bivand et al. 2017), *raster* (Hijmans 2017), *sf* (Pebesma 2018), *maptools* (Bivand and Lewin-Koh 2017) and *rgeos* (Bivand and Rundel 2017) packages.

## Results

### Daytime activity and distance traveled

**Fig. 1:**
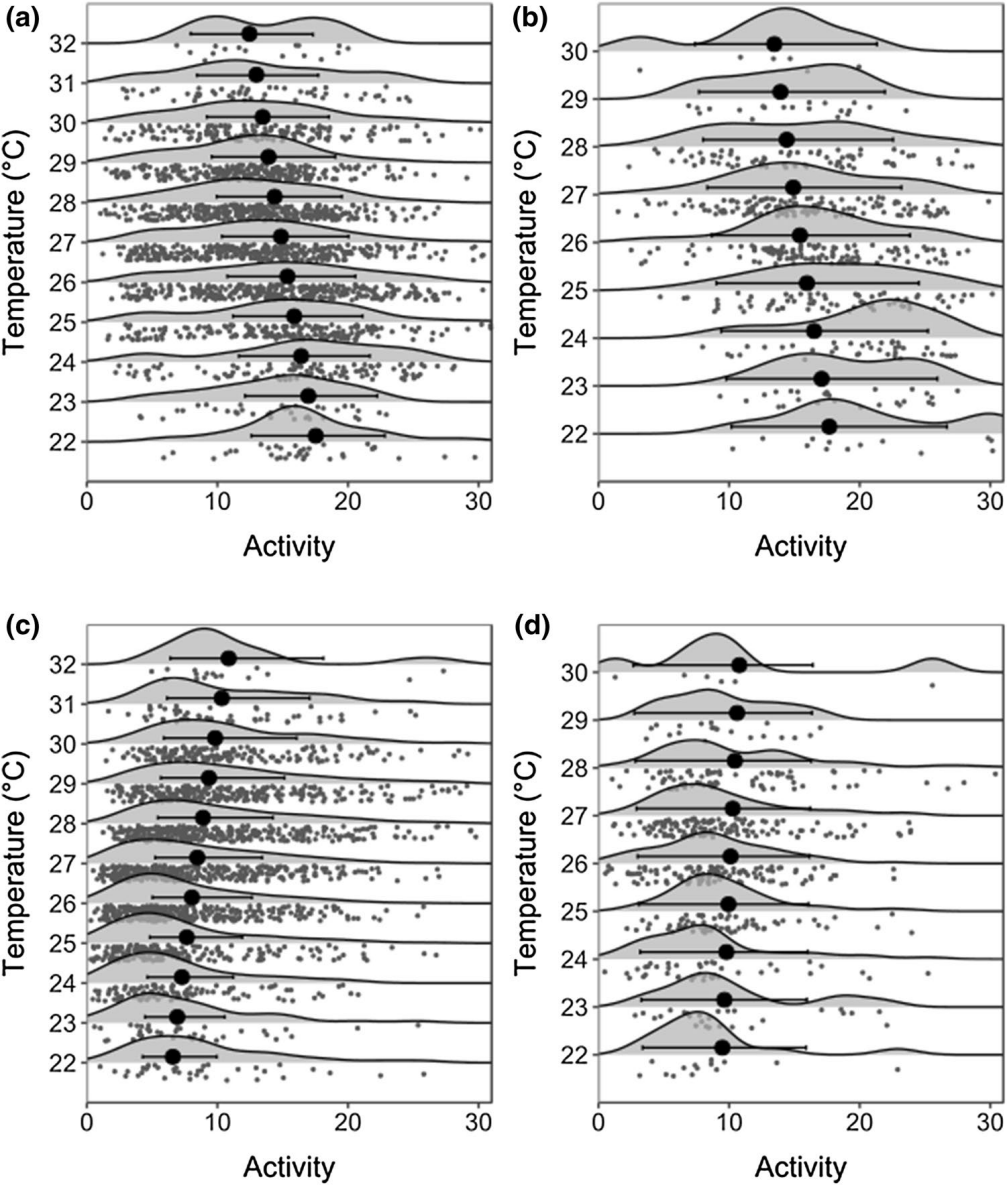
Relationships between African wild dog activity and maximum daytime temperature during **a** daylight hours outside the denning period **b** daylight hours inside the denning period **c** night-time hours outside the denning period and **d** night-time hours inside the denning period. Gray points represent the raw data, with the shaded curves representing the kernel density of the data in that 1 °C temperature band. Black circles represent model estimates, with black horizontal lines indicating standard errors. The model outputs were calculated using mean vales for rainfall and moonlight

Our analyses suggested that temperature, rainfall and denning status were all important predictors of African wild dog activity and distance traveled by day. During daylight hours, wild dogs were less active and traveled shorter distances on days when maximum daily temperatures were higher throughout both the denning and non-denning periods (Table 1, Fig. 1). Wild dogs also traveled less far when daily rainfall was higher during both denning and non-denning periods, but rainfall had a smaller effect, and was of lower importance, in the models of the denning period alone. An interaction between the effects of maximum temperature and rainfall was included in all top model sets; however, the effect size was small, with the 95% confidence interval crossing zero in all cases other than the models for activity in the denning and non-denning periods combined. Rainfall appears to slightly lessen the impact of high temperatures on daytime activity and distances traveled; however, this effect is less pronounced, or even reversed in the case of distance traveled, during the denning period (Table 1).

**Table 1:** Variables associated with wild dog activity and distance traveled during daylight hours

Period	Variable	Estimate	Activity Distance traveled (km)				
			95% CI	Importance (Δ < 2, Δ < 5)	Estimate	95% CI	Importance (Δ < 2, Δ < 5)
All	Intercept	3.58	3.4 to 3.8	(1, 3)	1.88	1.6 to 2.1	(4, 5)
	Maximum temperature (°C)	− 0.035	− 0.04 to − 0.03	1.00 (1, 3)	− 0.028	− 0.04 to − 0.02	1.00 (4,5)
	Daily rainfall (mm)	− 0.045	− 0.06 to − 0.03	1.00 (1,3)	− 0.012	− 0.05 to 0.02	0.95 (4, 4)
	Denning (Yes)	0.13	0.1 to 0.2	1.00 (1, 3)	0.54	− 0.1 to 1.01	1.00 (4, 5)
	Maximum temperature : Daily rainfall	0.0022	0.001 to 0.003	0.97 (1, 2)	0.001	− 0.0002 to 0.004	0.38 (2, 2)
	Maximum temperature : Denning (Yes)	0.000044	− 0.01 to 0.1	0.27 (0, 1)	− 0.028	− 0.5 to − 0.002	0.37 (2, 2)
Denning	Intercept	3.62	3.2 to 3.9	(3, 6)	2.47	1.7 to 3.3	(4, 10)
	Maximum temperature (°C)	− 0.034	− 0.05 to − 0.02	0.98 (3, 6)	− 0.049	− 0.08 to − 0.02	0.8 (3, 7)
	Daily rainfall (mm)	− 0.044	− 0.1 to 0.01	0.85 (3, 5)	− 0.012	− 0.05 to 0.02	0.38 (1, 6)
	Days at den	0.0016	− 0.001 to 0.002	0.95 (3, 6)	0.0059	− 0.001 to 0.01	1.00 (4, 10)
	Pup age	− 0.00051	− 0.001 to 0.0009	0.78 (2, 4)	− 0.0025	− 0.003 to − 0.001	0.60 (2, 7)
	Maximum temperature : Days at den	0.0034	0.002 to 0.005	0.22 (1, 2)	0.00075	0.0002 to 0.001	0.07 (0, 2)
	Maximum temperature : Rainfall	0.00015	− 0.00001 to 0.0003	0.48 (2, 2)	− 0.0056	− 0.01 to 0.001	0.10 (0, 2)

Averaged estimated effects of predictor variables on the daily distance traveled, and average activity, of wild dogs during dawn and dusk, for the whole dataset and whilst wild dogs were denning, estimated using generalised linear mixed effects models. As the residuals were gamma distributed an exponent of the values should be taken to obtain true estimates. Relative importance of each parameter is shown along with the number of models in the Δ < 2 and Δ < 5 model sets that contain each variable (*n*, *n*). Maximum temperature = maximum daily temperature (°C) during the 24-h period (dawn–dawn) and Daily rainfall = rainfall over 24-h period (mm). : denotes an interaction between two variables. Variables where no estimate is shown were Individual identity was included as a random variable

In line with hypothesis iii) wild dogs were more active and traveled further during the denning period (Table 1, Fig. 1). During the denning period, wild dogs were also more active and traveled longer distances when the pack had spent more days using a specific den site (Table 1). The interaction between days using a den site and maximum temperature was of low importance, included in 2 out of 6 of the top model sets, with a small positive interaction between maximum temperature and days spent at the den site. Pup age was included in the top model sets of activity and distance traveled during the denning period, and had a small negative effect on both.

### Night-time activity and distance traveled

Moonlight, temperature, rainfall and denning status were important predictors of wild dog activity and distances traveled by night. In line with hypothesis (i) packs increased night-time activity and distance traveled following hot days outside the denning period, (Denning (No) in Table 2). Contrary to our predictions, however, wild dogs did not significantly increase their nocturnal activity and distances traveled following hot days when denning (Table 2). The lack of increase in activity and distances traveled following hot days is indicated by negative interactions between the effects of temperature and denning on both distance traveled and activity levels in the models of denning and non-denning periods combined. This pattern is also shown by the 95% confidence intervals in the models of activity during the denning period crossing zero, and the negative relationship between temperature and distances traveled during the denning period (Table 2). Rainfall was of high importance for the denning and non-denning periods combined, being included in all top models for activity, and 3 out of 4 top models for distance traveled. Rainfall was included in a much lower proportion of the top models in the denning period, and the effect sizes were smaller. African wild dogs were more active at night when rainfall was higher.

**Table 2:** Variables associated with wild dog activity and distance traveled during night-time

Period	Variable	Activity			Distance traveled (km)		Importance (Δ < 2, Δ < 5)
		Estimate	95% CI	Importance (Δ < 2, Δ < 5)	Estimate	95% CI	
All	Intercept	0.71	0.5 to 0.9	(2,3)	0.71	0.3 to 1.0	(3, 5)
	Moonlight	0.039	0.03 to 0.05	1.00 (2, 3)	0.019	0.02 to 0.04	1.00 (3, 5)
	Maximum temperature (°C)	0.050	0.04 to 0.06	1.00 (2, 3)	0.033	0.02 to 0.04	0.97 (3, 5)
	Daily rainfall (mm)	0.051	0.01 to 0.08	1.00 (2, 3)	0.016	− 0.03 to 0.06	0.71 (2, 4)
	Denning (Yes)	0.72	0.2 to 1.2	1.00 (2, 3)	0.54	− 0.4 to1.6	1.00 (3, 5)
	Denning (Yes) : Maximum temperature	− 0.031	− 0.05 to − 0.02	0.73 (1, 2)	− 0.063	− 0.09 to − 0.03	0.53 (2, 2)
	Rainfall : Maximum temperature	− 0.0026	− 0.004 to − 0.001	0.85 (2, 2)	− 0.0018	− 0.004 to 0.0005	0.26 (1, 2)
Denning	Intercept	1.82	1.4 to 2.3	(2,8)	1.40	1.8 to 3.9	(6, 10)
	Moonlight	0.039	0.03–0.05	1.00 (2, 8)	0.0019	− 0.01 to 0.01	0.31 (1, 4)
	Maximum temperature (°C)	0.016	− 0.3 to 0.004	0.57 (1, 6)	− 0.013	− 0.05 to − 0.02	0.34 (2, 5)
	Daily rainfall (mm)	0.0025	− 0.023 to 0.028	0.29 (0, 4)	0.00058	− 0.04 to 0.04	0.20 (1, 3)
	Days at den	− 0.0094	− 0.03 to 0.007	0.31 (0, 4)	0.0015	− 0.01 to 0.01	0.38 (2, 3)
	Pup Age	0.00008	− 0.0002 to 0.002	0.19 (0, 2)	– 0.000015	− 0.002 to 0.002	0.15 (1,1 )
	Maximum temperature : Days at den	0.0014	0.0009 to 0.002	0.08 (0, 1)	*0.0028*	*0.001* *to* *0.004*	*0.01 (0, 0)*

Average estimated effects of predictor variables on the distance traveled and average activity of wild dogs from dusk to dawn, for the data as a whole and whilst the dogs were denning, estimated using generalised linear mixed effects models. As residuals were gamma distributed the exponent of the values should be taken to obtain true estimates. Relative importance of each parameter is shown along with the number of models in the Δ < 2 and Δ < 5 model sets that contain each variable (*n*,*n*). Italicized cells indicate variables that were not in any of the models with delta < 5. Maximum temperature = maximum daily temperature (°C) during the 24-h period (dawn–dawn) and Daily rainfall = rainfall over 24-h period (mm). : denotes an interaction between two variables. Variables where no estimate is shown were not in the Δ < 2 or Δ  <   5 model sets. Individual identity was included as a random variable

**Fig. 2:**
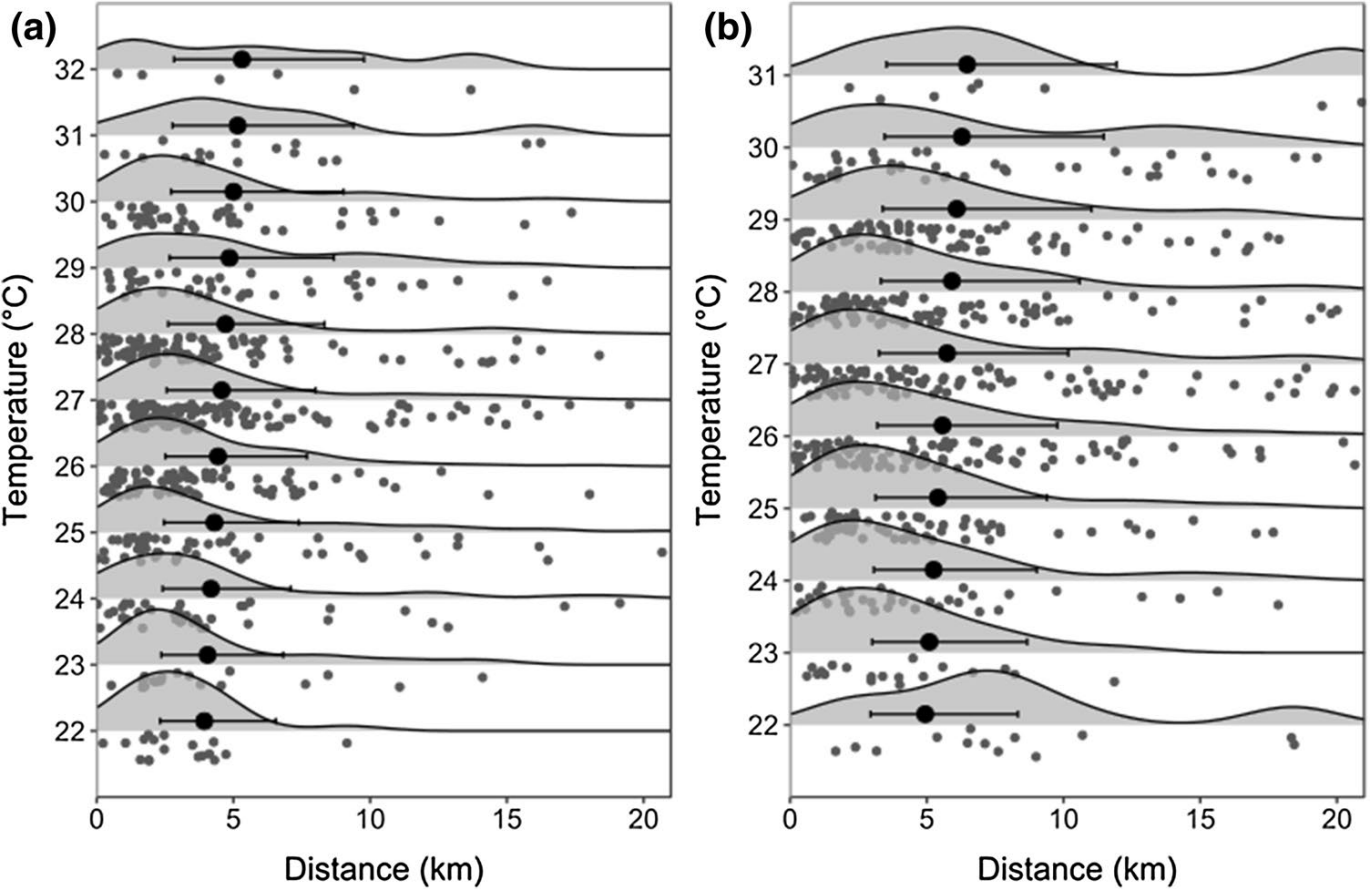
Relationship between daytime maximum temperature and subsequent night-time distances traveled by African wild dogs outside the denning period on nights with **a** low (0 h full moon equivalents) and **b** high (12-h full moon equivalents) levels of moonlight. Gray points represent the raw data, with the shaded curves representing the kernel density of the data in that 1 °C temperature band. Black circles represent model estimates, with black horizontal lines indicating the standard errors. The model outputs were calculated using mean daily rainfall

As predicted in hypothesis (ii) wild dogs were more active and traveled further when levels of moonlight were higher for denning and non-denning periods (Fig. 2, Table 2).

In line with hypothesis (iii) wild dogs were more active and traveled further during the denning period than outside it. Days at den and pup age were of relatively low importance as predictors of wild dog activity and distances traveled during denning, and the 95% confidence intervals associated with the effect sizes crossed zero in all cases. Within the models for activity in the denning period only, there was an interaction between the effects of maximum temperature and days spent at a particular den site included in the top model set; however, this variable was included in just 1 of the top 8 models and the effect size was small (Table 2).

### Activity and distance traveled over 24-h periods

**Fig. 3:**
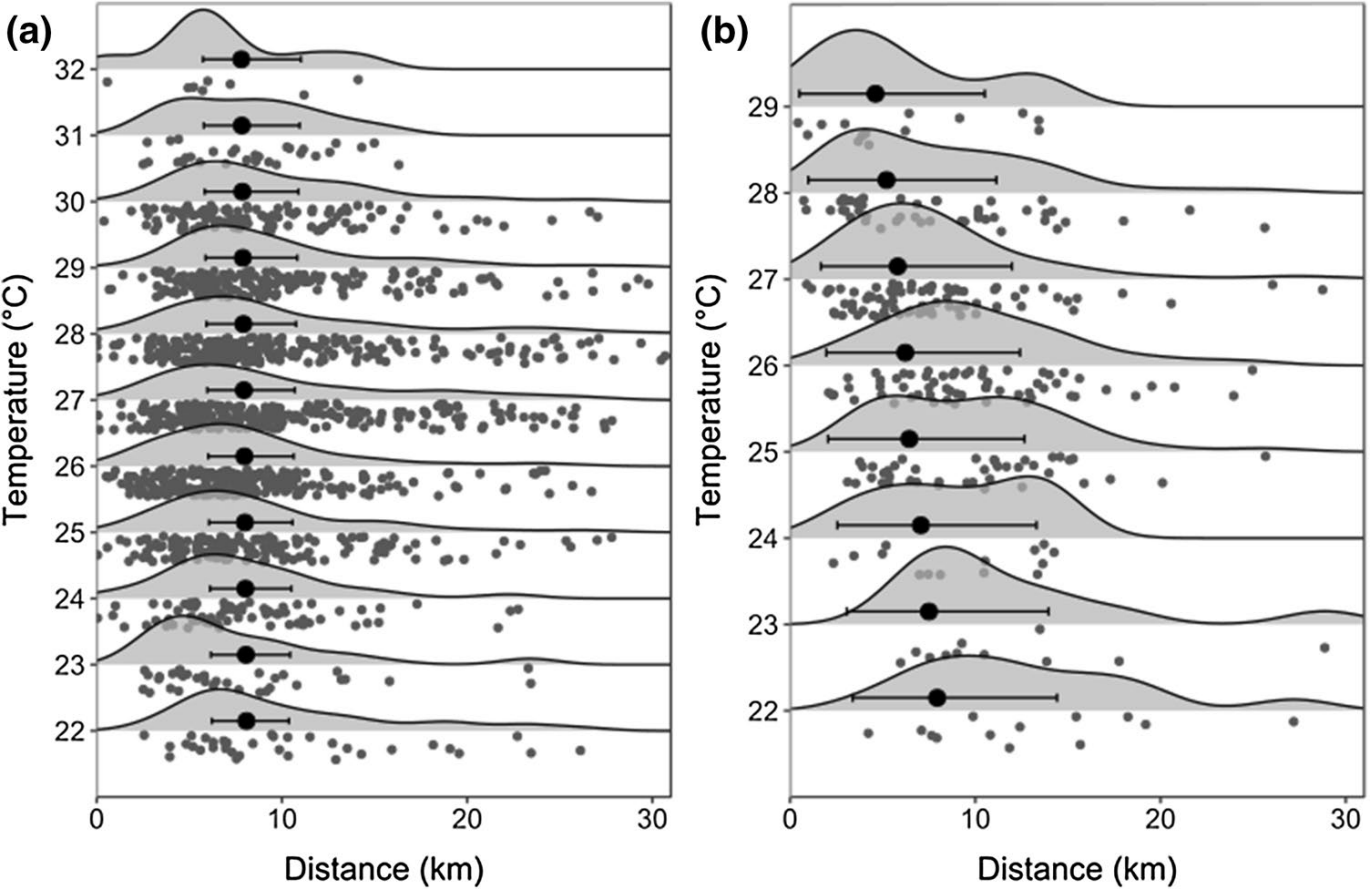
Relationship between total distance traveled across a 24-h period from dawn to dawn and temperature **a** outside and **b** inside the denning period. Gray points represent the raw data, with the shaded curves representing the kernel density of the data in that 1 °C temperature band. Black circles represent model estimates, with black horizontal lines indicating the standard errors. The model outputs were calculated for mean daily rainfall and moonlight

Denning, rainfall, temperature and moonlight were key predictors of activity and distances traveled across the 24-h period from dawn to dawn, for denning and non-denning periods combined. Contrasting with hypothesis (i) African wild dogs traveled less far and were less active on hot days both inside and outside the denning period. This negative effect of temperature, however, was greater inside the denning period than outside it, as indicated by the inclusion of a negative interaction between the effects of temperature and denning in the top models for both activity and distances traveled, and the importance of maximum temperature as a predictor of activity and distances traveled in the denning period (Table 3, Fig. 3). Despite being included in the top models, rainfall, days at the den site and pup age had little impact on activity and distances traveled, with confidence intervals for all estimates crossing zero.

**Table 3:** Variables associated with wild dog activity and distance traveled across a 24-h period

Period	Variable	Activity			Distance traveled (km)		
		Estimate	95% CI	Importance (Δ < 2, Δ < 5)	Estimate	95% CI	Importance (Δ < 2, Δ < 5)
All	Intercept	2.66	2.5 to 2.8	(3,5)	2.16	2.0 to 2.4	(4,12)
	Maximum temperature (°C)	− 0.0052	− 0.009 to − 0.002	0.95 (3, 4)	− 0.0035	− 0.008 to 0.007	0.69 (2, 6)
	Daily rainfall (mm)	0.0059	− 0.02 to 0.007	0.99 (3, 5)	0.0061	− 0.01 to 0.03	0.53 (2, 6)
	Moonlight	0.0043	0.003 to 0.006	1.00 (3, 5)	0.0072	0.003 to 0.01	0.87 (4, 9)
	Denning (Yes)	0.37	0.1 to 0.6	1.00 (3, 5)	0.63	− 0.3 to 1.5	0.77 (3, 8)
	Denning (Yes) : Maximum temperature	− 0.015	− 0.02 to − 0.009	0.66 (2, 3)	− 0.061	− 0.09 to -0.04	0.33 (2, 2)
	Maximum temperature : Rainfall	0.00074	0.0003 to 0.001	0.43 (1, 2)	− 0.00073	− 0.002 to 0.0007	0.11 (1, 1)
Denning	Intercept	2.90	2.6 to 3.2	(6,12)	2.71	2.0 to 3.4	(4, 16)
	Moonlight	0.006	0.003 to 0.009	0.85 (6, 9)	0.0042	0.0007 to 0.01	0.25 (0, 7)
	Maximum temperature (°C)	− 0.018	− 0.03 to − 0.007	0.78 (4, 10)	− 0.038	− 0.06 to − 0.01	0.61 (2, 10)
	Daily rainfall (mm)	− 0.00079	− 0.02 to 0.01	0.92 (6, 0)	0.0061	− 0.03 to 0.04	0.13 (0, 8)
	Days at den	− 0.0035	− 0.01 to 0.005	0.96 (6, 11)	0.0030	− 0.005 to 0.01	0.62 (3, 9)
	Pup Age	0.0036	− 0.0002 to 0.0009	0.57 (4, 7)	− 0.00021	− 0.003 to 0.0003	0.33 (1, 5)
	Maximum temperature : Days at den	0.00064	0.0005 to 0.0007	0.29 (2,2)	*0.0017*	*0.0009* *to* *0.003*	*0.02 (0, 0)*
	Maximum temperature : Rainfall	0.0001	0.0003 to 0.002	0.14 (1, 2)	− *0.0038*	− *0.01* *to* *0.002*	*0.01 (0, 0)*

Average estimated effects of predictor variables on the distance traveled and average activity of wild dogs across a 24-h period (sunrise–sunrise) estimated using generalized linear mixed effects models. The residuals of the models were gamma distributed and therefore the exponent of the values should be taken to obtain true estimates. Relative importance of each parameter is shown along with the number of models in the Δ < 2 and Δ < 5 model sets that contain each variable (*n*, *n*). Italicized cells indicate variables that were not in any of the models with delta < 5. Maximum temperature = maximum daily temperature (°C) during the 24-h period (dawn–dawn) and Daily rainfall = rainfall over 24-h period (mm). : denotes an interaction between two variables. Variables where no estimate is shown were not in the Δ<2 or Δ<5 model sets. Individual identity was included as a random variable

Across the denning and non-denning periods African wild dogs were more active, and traveled further, across a 24-h period when moonlight levels were higher, in line with hypothesis (ii). As predicted by hypothesis (iii) African wild dogs were more active and traveled further during the denning period compared to outside the denning period across a 24-h period (Table 3, Fig. 3). Top models for activity during the denning period included interactions between temperature and days spent at the den site, and temperature and rainfall. These were only included in 2 out of 12 top models, however, and had small positive effects.

### Projected changes in activity and distance traveled

Climate change projections suggested that the study site was expected to warm by between 1.6 and 3.9 degrees, and to experience lower rainfall levels by 2070 (Online Resource 11, Online Resource 12). In the best case climate scenario, wild dogs were predicted to reduce 24-h activity and distance traveled outside the denning period by 1%, reducing daytime activity by 5% but increasing nocturnal activity by 7%, and reducing daytime distance traveled by 4%, while increasing distance traveled at night by 6% (Table 4). During the denning period, however, the models predicted greater reductions in 24-h activity and distance traveled. African wild dogs were predicted to decrease 24-h activity and distances traveled by 4% and 8%, respectively, at average moonlight levels (Table 4).

**Table 4:** Predicted differences in mean activity (0–100) and total distances traveled (km) during the day, by night, and across 24 h from dawn to dawn between 2012 and 2016 and 2070 under the best and worst case climate scenarios

Time Period	Best case		Worst case	
	Activity	Distance (km)	Activity	Distance (km)
Not denning				
Day	− 0.71 (− 5%)	− 0.17 (− 4%)	− 1.00 (− 12%)	− 0.24 (− 10%)
Night	0.74 (7%)	0.31 (6%)	1.15 (18%)	0.50 (15%)
24 h	− 0.12 (− 1%)	− 0.04 (− 1%)	− 0.18 (− 2%)	− 0.05 (− 1%)
Denning				
Day	− 0.84 (− 5%)	− 0.27 (− 8%)	− 1.18 (− 13%)	− 0.35 (− 19%)
Night	0.29 (3%)	− 0.05 (− 2%)	0.43 (5%)	− 0.06 (− 5%)
24 h	− 0.37 (− 3%)	− 0.31 (− 6%)	− 0.53 (− 8%)	− 0.42 (− 14%)

Percentage change is shown in brackets. Best case is IPCC representation concentration pathway 2.6 and worst case is representation concentration pathway 8.5

Greater impacts were predicted in the worst case climate scenario, with wild dogs projected to reduce activity and distances traveled across a 24-h period by 2% outside the denning period, with a decrease in activity of 12% in the day and an increase of 18% at night. A decrease in daytime distances traveled of 10% was predicted, alongside a subsequent increase of 15% in night-time distance traveled. Inside the denning period, when wild dogs have first started using a den site, they were predicted to be 8% less active and travel distances 14% lower across a 24-h period than under current temperatures (Table 4).

## Discussion

We found that African wild dog activity and distance traveled were strongly associated with ambient temperature, moonlight, and pack reproductive state. As predicted, our results showed that on days with high maximum ambient temperatures, wild dogs showed lower daytime activity and moved shorter distances than they did on cooler days. In line with our hypotheses, high daytime temperatures were also associated with increased nocturnality. Outside the denning period, this increase in activity at night was nearly sufficient to balance lowered daytime activity, resulting in only slight reduction in activity and distance traveled at higher temperatures over a 24-h period. During the denning period, however, much more limited nocturnality meant that packs did not compensate for lost hunting activity during the day, with 24-h activity and distances traveled falling significantly when wild dogs had pups in the den.

Our finding that nocturnal activity was lower during the denning period contrasted with our prediction, which was based on an expectation that a means of coping with high daytime temperatures would be especially important when energetic demands are highest. This difference between prediction and observation may reflect packs’ need to guard their pups at night, when predators, such as lions (*Panthera leo)*, leopards (*Panthera pardus*), and hyaenas (*Crocuta crocuta*), are more active. Lower activity and distances traveled are likely to indicate that wild dogs are hunting less, which might in turn indicate lower food intake. Hence, a failure to compensate for lost hunting opportunities on hot days during the denning period may lead to decreased food intake for adults and pups alike, at a time when wild dogs face elevated energetic demands. This reduced food intake might, therefore, contribute to low pup survival at high ambient temperatures during the denning period. Similarly lower food intake in early life is likely to affect both the growth of the pups and their immune function (Moore et al. 2006), which may lead to higher mortality once they have left the den. Our findings, therefore, help to explain the lower survival of wild dogs pups raised at higher ambient temperatures (Woodroffe et al. 2017).

In contrast with our conclusions, Creel et al. (2016) suggested that high ambient temperatures might benefit wild dogs. They reported that wild dog hunts entailed shorter chases at higher temperatures, attributing this pattern to large-bodied prey overheating before their smaller-bodied predators. Chases, however, have been found to make up only around 8% of the distance covered by wild dogs in Northern Botswana (Hubel et al. 2016), a site where chase distances were significantly longer than those estimated for our study site (Woodroffe et al. 2007). The fact chases make up such a low percentage of distances covered by wild dogs would suggest that chases make up only a relatively small proportion of energy expenditure compared to searching for prey, similar to findings of studies into cheetah (*Acinonyx jubatus*) energetics (Scantlebury et al. 2014). If daytime hunting were more efficient on hotter days, as suggested by Creel et al. (2016), there would be no need for the subsequently increased activity at night described here, and no reduction in reproductive success at high ambient temperatures, as described by Woodroffe et al. The difference between this study and that of Creel et al. (2016) may also reflect differences in the size of prey species [10 kg (main prey species dik-dik and impala), Woodroffe et al. 2007, vs 88 kg mean mass (main prey species wildebeest and impala), Creel et al. 2016] and the more open habitat found in Creel et al’s (2016) study site that could potentially facilitate longer chases.

Wild dogs’ nocturnal activity and ranging behavior was restricted by levels of moonlight (also see Pole 2000; Cozzi et al. 2012; Rasmussen and Macdonald 2012). Rasmussen and Macdonald (2012) suggested that wild dogs increased their night-time activity in response to human presence, suggesting that packs might likewise be able to increase their nocturnality in response to high ambient temperatures. However, Rasmussen and Macdonald (2012) also found that nocturnality was also found to be restricted by low levels of moonlight. As nearly half of nights have moonlight levels of less than 25% full-moon-night equivalents (Online Resource 13), moonlight appears to be a major constraint on nocturnal wild dog activity.

Wild dogs’ tendency to avoid hunting on moonless nights might reflect limited visual acuity at low light levels (Jacobs 1993). Poor nocturnal vision would make both hunting and avoidance of competitors challenging, as wild dogs may be less able to detect lions and hyaenas during nights with less light, resulting in greater levels of kleptoparasitism and mortality. However, wild dogs’ nocturnality was also largely restricted to moonlit nights at a site with very low lion and hyaena numbers (Pole 2000), suggesting that wild dogs’ reliance on moonlight may be related to their own hunting ability at low light levels, rather than predator avoidance.

Wild dogs were less active and traveled less far in the day, and traveled further at night, when the weather was wetter, likely due to sheltering from the rain. Interaction terms indicate that rainfall reduces the impact of high temperatures a small amount. The decreased response to high temperatures observed at higher rainfall levels might reflect the lower ambient temperatures observed directly after rain (Woodroffe et al. 2017), greater cloud cover, or access to standing water facilitating heat loss, meaning that wild dogs’ activity is less restricted by high temperatures on days where it rains. This impact of rainfall on activity and distances traveled was far less marked in the denning period, which may reflect increased energetic demands on the dogs when they have pups to feed, forcing them to hunt even in sub-optimal weather.

Future projections suggest that wild dogs will be less active and travel less far under future climate change, particularly in the denning period. Recent climate assessments have suggested that the best case scenario is unlikely, and therefore, future increases in temperature are likely to be higher than those used in our best case scenario predictions (Cox et al. 2018). This would mean that when wild dogs are denning average decreases in activity of greater than the 3% per 24 h and decreases in distances traveled of greater than 6% per 24 h, as predicted in the best case scenario, are likely by the year 2070. As demographic effects are already apparent at high temperatures under the current climate regime (Woodroffe et al. 2017), the impact of the consistently higher temperatures projected for 2070 on wild dog demography is likely to be marked. As African wild dogs’ hunting strategy relies on covering large distances to consume enough prey to maintain their energy balance (Hubel et al. 2016), they potentially have greater energy expenditure than many other species (Gorman et al. 1998). Reduced activity and distances traveled at high temperatures are likely to have an impact on food intake, and hence may exacerbate the impacts of temperature on recruitment already observed in the field (Woodroffe et al. 2017). Outside the denning period, while 24-h activity and distance traveled might change little, the shift from day-time to night-time hunting, with around 5–10% of their activity shifting from day to night, might decrease wild dog hunting success as a result of a greater percentage of hunts occurring at low light levels, as well as putting wild dogs at greater risk of predation by lions and hyaenas.

Our projections may under-estimate the impact of climate change, since they assume there are no restrictions on wild dogs’ ability to increase their nocturnal activity outside the denning period. However, wild dogs’ nocturnality was heavily constrained by the availability of moonlight. Projections were modeled at average moonlight levels; however, there can be periods of up to 18 consecutive days where moonlight levels are lower than this. At high temperatures during these periods of low moonlight, wild dogs’ nocturnal activity will be even further limited, and this may impose further reductions in food intake for individuals across those periods. Although wild dogs might experience relatively high food intake during moonlit periods, they would be unable to maintain this intake through periods without moonlight, because they do not cache their food. Hence, low moonlight levels are likely to place them under more energetic stress in combination with hot weather, compared to periods of high levels of moonlight. This change could result in lowered food intake at higher temperatures when moonlight levels are low, which may have effects on adult condition, and even mortality.

Our findings highlight the constraints to climate change adaptation in African wild dogs. There is little opportunity for wild dogs to adapt to rising temperatures in space, since their distribution is already limited by habitat loss and human activity (Woodroffe and Sillero-Zubiri 2012). For wild dog ranges to expand into new areas, there would need to be an extensive programme of habitat restoration and translocations (Gusset et al. 2008). As they already breed at the coolest period of the year where this period is predictable (McNutt et al. 2017), there is no opportunity for shifts in the timing of breeding to compensate for rising temperatures. Previous studies have suggested that large mammals like the African wild dog are unlikely to be able to adapt to rising temperatures through evolutionary change, as their long generation times mean that climate change is likely to outpace the species’ rate of evolution (Fuller et al. 2016). Since wild dogs have little potential for adaptation in time and space, and are limited in their rate of evolutionary change by long generation times, this leaves behavioral adaptation as one of the most plausible forms of climate change adaptation. However, our findings suggest that a shift to increased nocturnal hunting would be severely constrained in the African wild dog, because both moonlight and the need to guard pups during the denning period will remain fixed as temperatures rise. Consequently, wild dogs may not be behaviorally flexible enough to enable them to compensate sufficiently as temperatures continue to rise, contrasting with the pattern reported from other species, which have been observed shifting activity to cooler times and microclimates in response to higher temperatures (Hetem et al. 2012; Cunningham et al. 2015). These findings have clear implications for the future conservation of African wild dogs, as it may be necessary to focus conservation efforts on areas predicted to experience less warming in the future, for example, the southernmost parts of Africa or high altitude areas, to protect the species from extinction.

### Wider implications

Behavioral plasticity is likely to be a key determinant of the severity of climate change impacts across a wide variety of species. Mammals have some of the most complex behaviors of any taxa, and behavioral plasticity is likely to play an important part in their responses to climate change (McCain and King 2014). Such flexibility may be particularly significant for crepuscular species, since, as temperatures rise, each day will include fewer hours when low ambient temperatures coincide with high light levels. Behavior changes at higher temperatures are likely to be key in determining species’ climate change responses, and ultimately impacts of climate change on the species’ viability in the future. It is vital that crepuscular species’ ability to become active at night is investigated in future research.

The ability of species to shift their behavior in response to high temperatures is likely to be an important determinant of the extent to which they can adapt to rising temperatures. However, thermoregulatory behaviors must be traded off against other behaviors, such as foraging (Cunningham et al. 2015), and have been found to impact reproduction in some species (Cunningham et al. 2013). In the African wild dog, the thermoregulatory behavior of lower activity during the day on hot days, coupled with their constrained behavioral shift to nocturnality during the denning period, is likely to result in lower pup survival. It is important that such trade-offs are identified, and their effects on population dynamics and viability established, for other species. To identify these impacts, long-term, detailed, studies of species are essential. Behavioral shifts need to be incorporated into vulnerability assessments of species through including the behavioral plasticity of species into trait-based assessments where this information is available. For species with high conservation priority, mechanistic, species-specific assessments which incorporate detailed behavioral responses to climate change are likely to be most appropriate in informing future conservation actions.

## Electronic supplementary material

Below is the link to the electronic supplementary material.

Supplementary material 1 (DOCX 2264 kb)

## References

[CR1] Barton K (2002). MuMIn: Multi-model inference: a practical information theoretic approach.

[CR2] Bates D, Maechler M, Bolker B, Walker S (2015). Fitting linear mixed-effects models using lme4. J Stat Softw.

[CR3] Beever EA, Brussard PF, Berger J (2003). Patterns of apparent extirpation among isolated populations of pikas (Ochotona Princeps) in the Great Basin. J Mammal.

[CR4] Bellard C, Bertelsmeier C, Leadley P (2012). Impacts of climate change on the future of biodiversity. Ecol Lett.

[CR5] Bivand R, Lewin-Koh N, Pebesma EJ, Archer E, Baddeley A, Bibiko HJ, Dray S, Forrest D, Friendly M, Giraudoux P, Golicher D, Rubio VG, Hausmann P, Hufthammer KO, Jagger T, Luque SP, MacQueen D, Niccolai A, Short T, Stabler B, Turner R (2017) Package: ‘Maptools’. R package version 0.9-4. http://CRAN.R-project.org/package=maptools

[CR6] Bivand R, Rundel C (2017) Rgeos: interface to geometry engine-open source (‘GEOS’) R package version: 04-2. https://cran.r-project.org/package=rgeo

[CR7] Bivand R, Keitt T, Rowlingson B (2017) Rgdal: bindings for the “Geospatial” data abstraction library. R package version: 1.3-6. https://cran.r-project.org/package=rgdal

[CR8] Briscoe NJ, Handasyde KA, Griffiths SR (2014). Tree-hugging koalas demonstrate a novel thermoregulatory mechanism for arboreal mammals Tree-hugging koalas demonstrate a novel thermoregulatory mechanism for arboreal mammals. Biol Lett.

[CR9] Burnham KP, Anderson DR (2002). Burnham KP Model selection and multimodel inference: a practical
information-theoretic approach.

[CR10] Caylor KK, Gitonga J, Martins DJ (2016) Meteorological and hydrological dataset. http://www.mpala.org/Publications.php

[CR11] Cox PM, Huntingford C, Williamson MS (2018). Emergent constraint on equilibrium climate sensitivity from global temperature variability. Nature.

[CR12] Cozzi G, Broekhuis F, McNutt J (2012). Fear of the dark or dinner by moonlight ? Reduced temporal partitioning among Africa’ s large carnivores. Ecology.

[CR13] Creel S, Creel NM (2002). The African wild dog: behavior, ecology, and conservation.

[CR14] Creel S, Creel NM, Creel AM, Creel BM (2016). Hunting on a hot day: effects of temperature on interactions between African wild dogs and their prey. Ecology.

[CR15] Cunningham SJ, Martin RO, Hojem CL, Hockey PA (2013). Temperatures in excess of critical thresholds threaten nestling growth and survival in a rapidly-warming arid savanna: a study of common Fiscals. PLoS One.

[CR16] Cunningham SJ, Martin RO, Hockey PA (2015). Can behaviour buffer the impacts of climate change on an arid-zone bird?. Ostrich J African Ornithol.

[CR17] du Plessis KL, Martin RO, Hockey PA (2012). The costs of keeping cool in a warming world: implications of high temperatures for foraging, thermoregulation and body condition of an arid-zone bird. Glob Chang Biol.

[CR18] Foden WB, Butchart SHM, Stuart SN (2013). Identifying the world’s most climate change vulnerable species: a systematic trait-based assessment of all birds, amphibians and corals. PLoS One.

[CR19] Fuller A, Mitchell D, Maloney SK, Hetem RS (2016). Towards a mechanistic understanding of the responses of large terrestrial mammals to heat and aridity associated with climate change. Clim Chang Responses.

[CR20] Gorman ML, Mills MG, Raath JP, Speakman JR (1998). High hunting costs make African wild dogs vulnerable to kleptoparasitism by hyaenas. Nature.

[CR21] Gusset M, Ryan SJ, Hofmeyr M (2008). Efforts going to the dogs? Evaluating attempts to re-introduce endangered wild dogs in South Africa. J Appl Ecol.

[CR22] Harrison XA, Donaldson L, Correa-Cano ME (2018). A brief introduction to mixed effects modelling and multi-model inference in ecology. PeerJ.

[CR23] Hetem RS, Strauss WM, Fick LG (2012). Activity re-assignment and microclimate selection of free-living Arabian oryx: responses that could minimise the effects of climate change on homeostasis?. Zoology.

[CR24] Hijmans R (2017) Raster: geographic data analysis and modeling. R package version 2.8-4. https://cran.r-project.org/package=raster

[CR25] Hijmans RJ, Cameron SE, Parra JL (2005). Very high resolution interpolated climate surfaces for global land areas. Int J Climatol.

[CR26] Hubel TY, Myatt JP, Jordan NR (2016). Energy cost and return for hunting in African wild dogs and cheetahs. Nat Commun.

[CR27] Huey RB, Kearney MR, Krockenberger A (2012). Predicting organismal vulnerability to climate warming: roles of behaviour, physiology and adaptation. Philos Trans R Soc Lond B Biol Sci.

[CR56] IPCC (2014) Climate change 2014: synthesis report. Contribution of working groups I, II and III to the fifth assessment report of the intergovernmental panel on climate change. In: Pachauri RK, Meyer LA (eds). IPCC, Geneva, Switzerland, 151 pp

[CR28] Jacobs GH (1993). The distribution and nature of colour vision among the mammals. Biol Rev.

[CR29] Kearney M, Shine R, Porter WP (2009). The potential for behavioral thermoregulation to buffer “cold-blooded” animals against climate warming. Proc Natl Acad Sci USA.

[CR30] Kearney MR, Wintle BA, Porter WP (2010). Correlative and mechanistic models of species distribution provide congruent forecasts under climate change. Conserv Lett.

[CR31] Malcolm JR, Marten K (1982) Natural selection and the communal rearing of pups in African Wild Dogs (Lycaon pictus). Behav Ecol Sociobiol 10(1): 1–13. JSTOR, www.jstor.org/stable/4599454. Accessed 15 Sep 2016

[CR32] Martin RO, Cunningham SJ, Hockey PA (2015). Elevated temperatures drive fine-scale patterns of habitat use in a savanna bird community. Ostrich.

[CR33] McCain CM, King SRB (2014). Body size and activity times mediate mammalian responses to climate change. Glob Chang Biol.

[CR34] McNutt JW, Groom R, Woodroffe R (2017) High ambient temperatures provide an adaptive explanation for seasonal reproduction in a tropical mammal. (In Review)

[CR35] Moore SE, Collinson AC, Tamba N’Gom P (2006). Early immunological development and mortality from infectious disease in later life. Proc Nutr Soc.

[CR36] Pacifici M, Foden WB, Visconti P (2015). Assessing species vulnerability to climate change. Nat Clim Chang.

[CR37] Parmesan C (1996). Climate and species’ range. Nature.

[CR38] Parmesan C (2006). Ecological and evolutionary responses to recent climate change. Annu Rev Ecol Syst.

[CR39] Parmesan C, Yohe G (2003). A globally coherent fingerprint of climate change impacts across natural systems. Nature.

[CR40] Pebesma E (2018) sf: Simple features for R. R package version: 0.7-2. https://cran.r-project.org/package=sf

[CR41] Pole A (2000) The behaviour and ecology of african wild dogs, Lycaon Pictus, in an environment with reduced competitor density. Doctoral Thesis, Aberdeen University, Aberdeen

[CR42] Pounds JA, Bustamante MR, Coloma LA (2006). Widespread amphibian extinctions from epidemic disease driven by global warming. Nature.

[CR43] Rasmussen GSA, Macdonald DW (2012). Masking of the zeitgeber: african wild dogs mitigate persecution by balancing time. J Zool.

[CR44] Scantlebury DM, Mills MGL, Wilson RP (2014). Flexible energetics of cheetah hunting strategies provide resistance against kleptoparasitism. Science.

[CR45] Sinervo B, Mendez-De-La-Cruz F, Miles (2010). Rosion of lizard diversity by climate change and altered thermal niches. Science.

[CR46] R Core Team (2018) R: A language and environment for statistical computing. R Foundation for Statistical Computing, Vienna, Austria. http://www.R-project.org/

[CR47] Tingley MW, Koo MS, Moritz C (2012). The push and pull of climate change causes heterogeneous shifts in avian elevational ranges. Glob Chang Biol.

[CR48] Turbill C, Prior S (2016). Thermal climate-linked variation in annual survival rate of hibernating rodents: shorter winter dormancy and lower survival in warmer climates. Funct Ecol.

[CR49] Bogale G, Awlachew SB, Dinku T, Nyenzi BS, Moges S, Sileshi I, Ogalo L (2011) Economic commission for Africa economic commission for Africa united nations economic commission for Africa African climate policy centre assessment of Africa's climatic records and recording networks including strategic for rescuing of climatic data united nations economic commission for Africa African climate policy centre working paper 3. An assessment of Africa's climate observing networks and data including strategies for rescuing of climatic data. ​Adis Ababa

[CR50] Walther G-R, Post E, Convey P (2002). Ecological responses to recent climate change. Nature.

[CR51] Woodroffe R (2010). Ranging behaviour of African wild dog packs in a human-dominated landscape. J Zool.

[CR52] Woodroffe R (2011). Demography of a recovering African wild dog (Lycaon pictus) population. J Mammal.

[CR53] Woodroffe R, Sillero-zubiri C (2012) *Lycaon pictus*. IUCN red list threat species Version 2016-1. http://iucnrle.org

[CR54] Woodroffe R, Lindsey PA, Romañach SS, old Ranah SMK (2007). African wild dogs (*Lycaon pictus*) can subsist on small prey: implications for conservation. J Mamm.

[CR55] Woodroffe R, Groom R, McNutt JW (2017). Hot dogs: High ambient temperatures impact reproductive success in a tropical carnivore. J Anim Ecol.

