# Supplementary material for: Coping with climate change: limited behavioral responses to hot weather in a tropical carnivore
Source: Oecologia. 2019 Feb 10;189(3):587–99. doi: 10.1007/s00442-018-04329-1 (PMC6418050; doi:10.1007/s00442-018-04329-1)
Supplement: Supplementary file 1 — Supplementary material 1 (DOCX 2264 kb) [file 442_2018_4329_MOESM1_ESM.docx]

***Electronic Supplemental Material for Coping with climate change: limited behavioural responses to hot weather in a tropical carnivore***

Authors: Daniella Rabaiotti^1,2^ and Rosie Woodroffe^1^

1. Institute of Zoology, Zoological Society of London, Regents Park, London, NW1 4RY

2. Centre for Biodiversity and Environment Research, Department of Genetics, Evolution and Environment, Division of Biosciences, University College London, Gower Street, London, WC1H 0AG

Corresponding Author: Daniella Rabaiotti, Institute of Zoology, Zoological Society of London, Regents Park, London, NW1 4RY.

Email: Daniella.Rabaiotti@ioz.ac.uk

Phone: + 44 7968018087

**Online Resource 1** – Monitoring periods for GPS-collared African wild dogs at our Kenya study site. Monitoring started when GPS-collars were fitted, and ended when the collar was removed, the battery expired, or contact was lost. Two dogs – WDF120 and WDF126 changed packs during monitoring, moving to the Truant pack on the 29/09/14 and the Toucan pack on the 29/03/15 respectively.

|  |  |  | **GPS-collar monitoring** | |  |
| --- | --- | --- | --- | --- | --- |
| **ID** | **Pack 1** | **Sex** | *start* | *end* | *Number of days* |
| WDM91 | Tui’s | M | 27 Jul 13 | 01-Aug-14 | 370 |
| WDF96 | Loisaba | F | 15 Mar 12 | 31-Jan-13 | 322 |
| WDM97 | Rat | M | 27 Apr 11 | 27-May-11 | 30 |
| WDF105 | Loisaba | F | 14 Sep 11 | 25-Nov-11 | 72 |
| WDF109 | Lebai | F | 07 Apr 11 | 12-Aug-11 | 127 |
| WDM111 | Loisaba | M | 02 Mar 12 | 10-Jun-12 | 100 |
| WDM112 | Tui’s | M | 8 Mar 12 | 08-Dec-12 | 275 |
| WDM118 | Bahati | M | 24 Jul 13 | 25-Jan-14 | 185 |
| WDM119 | Loisaba | M | 3 Aug 13 | 30-Sep-14 | 423 |
| WDF120 | Tui’s | F | 31 Jul 14 | 28-Nov-14 | 120 |
| WDF123 | Crocodile | F | 23 Apr 14 | 23-Jan-15 | 275 |
| WDF126 | Katu | F | 1 Aug 14 | 03-May-15 | 275 |
| WDF130 | Ol Pejeta | F | 27 Aug 14 | 14-Dec-14 | 109 |
| WDM131 | Crocodile | M | 24 Apr 15 | 13-Jun-15 | 50 |
| WDM132 | Bahati | M | 25 Apr 15 | 21-Jun-15 | 57 |

**Online Resource 2 -** Times of GPS fixes of each wild dog GPS-collar used in the study – X denotes a GPS fix that was included in the distance analyses. ○ denotes a GPS fix that was discarded from the dataset. Day was considered to be 6:00 or 6:30 to 18:00. Night was considered to be 18:00 to 06:30. Dogs not used in the night analysis due to having too few nocturnal GPS fixes are highlighted in grey.

| **WD** | **01:00** | **02:15** | **03:30** | **05:00** | **06:00** | **06:30** | **07:00** | **07:30** | **08:00** | **10:00** | **13:00** | **16:00** | **18:00** | **18:30** | **19:30** | **20:00** | **20:45** | **22:00** | **23:30** |
| --- | --- | --- | --- | --- | --- | --- | --- | --- | --- | --- | --- | --- | --- | --- | --- | --- | --- | --- | --- |
| WDM91 | X |  | ○ |  |  | X | ○ |  | X |  | X | ○ | X |  | X |  |  | ○ |  |
| WDF96 | X |  |  |  |  | X | ○ | ○ | X |  | X | ○ | X | ○ | X |  |  |  |  |
| WDM97 | X |  |  |  |  | X |  |  | X |  | X | ○ | X |  | X |  |  |  |  |
| WDF105 | X |  |  |  |  | X | ○ | ○ | X |  | X |  | X | ○ | X |  |  |  |  |
| WDF109 | X |  |  |  |  | X |  |  | X |  | X |  | X |  | X |  |  |  |  |
| WDM111 | X |  |  |  |  | X | ○ | ○ | X |  | X |  | X | ○ | X |  |  |  |  |
| WDM112 | X |  |  |  |  | X | ○ | ○ | X |  | X |  | X | ○ | X |  |  |  |  |
| WDM118 | X |  | ○ |  |  | X | ○ |  | X |  | X |  | X |  | X |  |  | ○ |  |
| WDM119 | X |  | ○ |  |  | X | ○ |  | X |  | X |  | X |  | X |  |  | ○ |  |
| WDF120 | X |  | ○ |  |  | X | ○ |  | X |  | X |  | X |  | X |  |  | ○ |  |
| WDF123 | X |  |  |  |  | X | ○ | ○ | X |  | X |  | X | ○ | X |  |  |  |  |
| WDF126 | X |  | ○ |  |  | X | ○ |  | X |  | X |  | X |  | X |  |  | ○ |  |
| WDF130 | X |  | ○ |  |  | X | ○ |  | X |  | X |  | X |  | X |  |  | ○ |  |
| WDM131 | X | ○ | ○ | ○ |  | X | ○ |  | X |  | X |  | X |  | X |  | ○ | ○ | ○ |
| WDM132 | X | ○ | ○ | ○ |  | X | ○ |  | X |  | X |  | X |  | X |  | ○ | ○ | ○ |

**Online Resource 3 -** Number of days and nights of data included in the analyses per GPS-collared wild dog

| Individual identity | Daytime activity | Daytime distance travelled | Night-time activity | Night-time distance travelled | 24 hour activity | 24 hour distance travelled |
| --- | --- | --- | --- | --- | --- | --- |
| WDM91 | 332 | 269 | 333 | 277 | 332 | 244 |
| WDF96 | 260 | 195 | 261 | 200 | 260 | 152 |
| WDM97 | 30 | 22 | 23 | 21 | 23 | 18 |
| WDF105 | 71 | 56 | 72 | 54 | 71 | 34 |
| WDF109 | 127 | 79 | 127 | 85 | 122 | 57 |
| WDM111 | 4 | 92 | 5 | 87 | 4 | 49 |
| WDM112 | 274 | 187 | 275 | 187 | 274 | 133 |
| WDM118 | 13 | 166 | 13 | 164 | 13 | 145 |
| WDM119 | 349 | 275 | 349 | 274 | 348 | 233 |
| WDF120 | 120 | 98 | 121 | 91 | 120 | 66 |
| WDF123 | 273 | 131 | 273 | 139 | 273 | 74 |
| WDF126 | 273 | 273 | 273 | 252 | 273 | 212 |
| WDF130 | 148 | 134 | 148 | 131 | 148 | 120 |
| WDM131 | 108 | 70 | 109 | 59 | 108 | 35 |
| WDM132 | 50 | 36 | 50 | 37 | 50 | 23 |
| Total | **2432** | **2089** | **2432** | **2058** | **2419** | **1595** |

**Online Resource 4** – Reasons for including explanatory variables and expected outcomes. + indicates an expected increase in activity and distance travelled and – indicates an expected decrease.

| **Variable** | **Reason** | **Expected outcome** | |
| --- | --- | --- | --- |
|  |  | **Day** | **Night** |
| *Daily maximum temperature* | Maximum temperature was chosen as it has been found to be the ecologically relevant temperature variable impacting wild dog behaviour in a number of other studies which have also included other variables such as average daily temperature (Woodroffe et al 2011a, Woodroffe, Groom and McNutt 2017). Woodroffe, Groom and McNutt (2017) found that high daily maximum temperatures during the denning period were associated with low pup survival. | - | + |
| *Daily rainfall* | Woodroffe, Groom and McNutt (2017) found that rainfall had an effect on pup survival. We wanted to investigate if this was a result of impacts on adult behavior. | Due to inconsistent effects of rainfall on pup survival we were unsure of how rainfall would impact wild dog behavior. | |
| *Level of moonlight* | Cozzi *et al* (2012) found a positive relationship between night-time wild dog activity and levels of moonlight. Including this variable allowed us to investigate whether constraints of moonlight levels prevent wild dogs from hunting at night. | NA | + |
| *Denning* | Woodroffe, Groom and McNutt (2017) found that wild dogs have greater energy demands when they are denning, and travelled further during a 24 hour period. | + | + |
| *Age of pups* | As pups grow they require more food and therefore greater energy expenditure by the adults. | + | + |
| *Days at den site* | During the denning period the pack typically moves its den site a number of times in response to threats and prey depletion around the area of the den. Ford et al found that as wild dogs spend time at a den site prey becomes locally depleted (Ford et al., 2015), which forces them to travel further for food (Woodroffe, Groom & McNutt, 2017). | + | + |
| *Daily maximum temperature * Daily rainfall* | Rainfall often mediates the impacts of high temperatures. | + | - |
| *Daily maximum temperature * Denning* | To test if responses differ significantly inside compared to outside the denning period | Unknown | Unknown |
| *Daily maximum temperature * Days at den site* | Dogs may need to increase their activity on hot days as prey becomes locally depleted at a den site. | + | + |

**Online Resource 5** – Diagram and description of moonlight calculations. Circles indicate the moon, ↑ indicates moonrise, ↓ indicates moonset. Numbers correspond to scenarios in the below table.

Day 1

Night

1

1

2

2

3

3

4

4

Day 2

Sunset

Sunrise 2

Sunrise 1

| **Scenario number** | **Scenario** | **Calculation for hours of moonlight** | **Calculation to give final moonlight value** |
| --- | --- | --- | --- |
| 1 | Moon rises and sets the day before. | 0 moonlight. | Multiply by percentage illumination of the moon that night |
| 2 | Moon rises the previous day and sets during the night. | moonset - sunset |  |
| 3 | Moon rises and sets during the night | moonset - moonrise |  |
| 4 | Moon rises at night and sets the next day | sunrise 2 - moonrise |  |

**Online Resource 6**: Histograms of model residuals

| Night-time activity (denning and non-denning) | Daytime activity (denning and nondenning) |
| --- | --- |
| 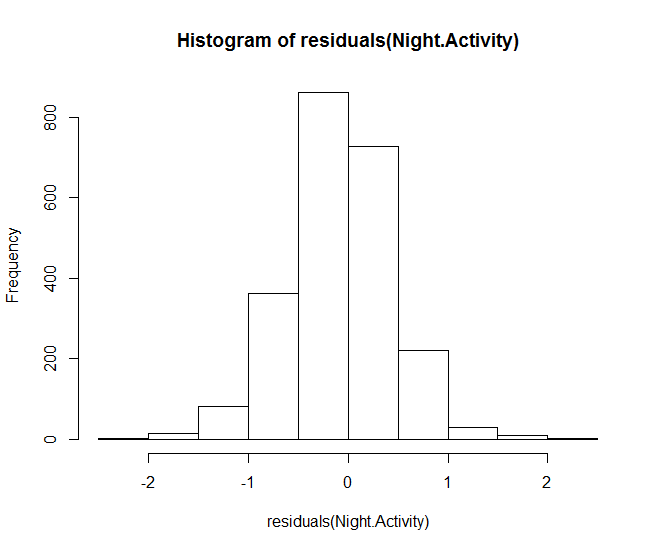 | 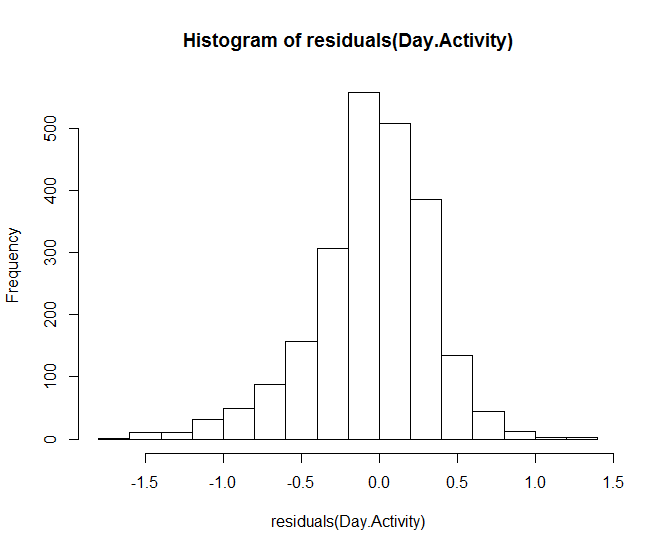 |
| Day-time activity (denning) | Night-time activity (denning) |
| 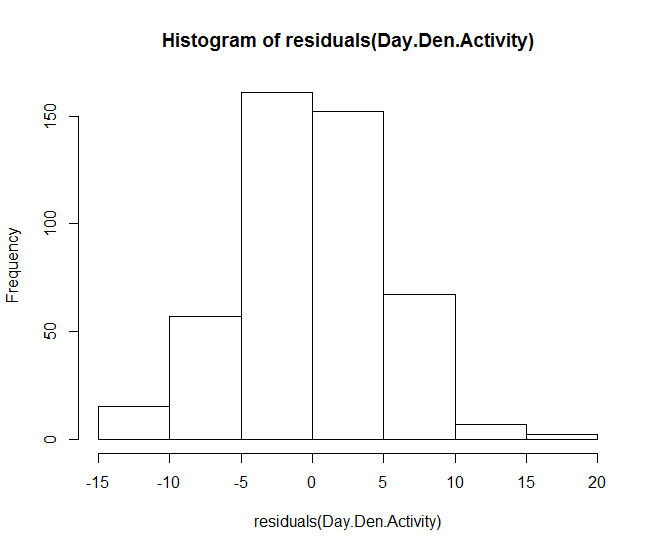 | 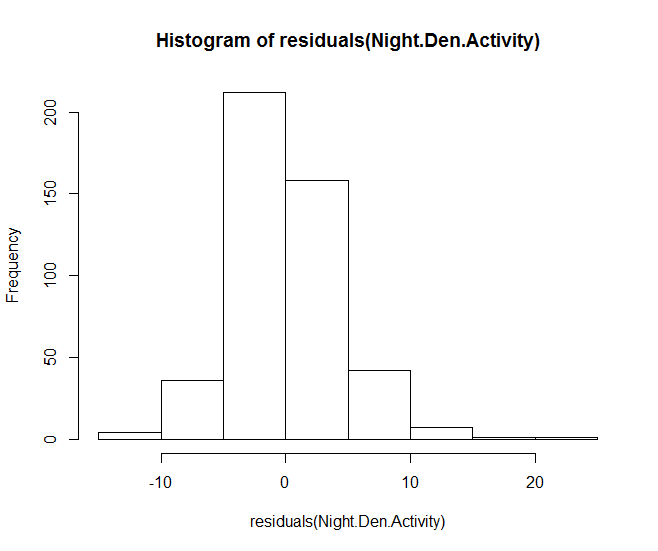 |
| 24 hour activity (denning and non-denning) | 24 hour activity (denning) |
| 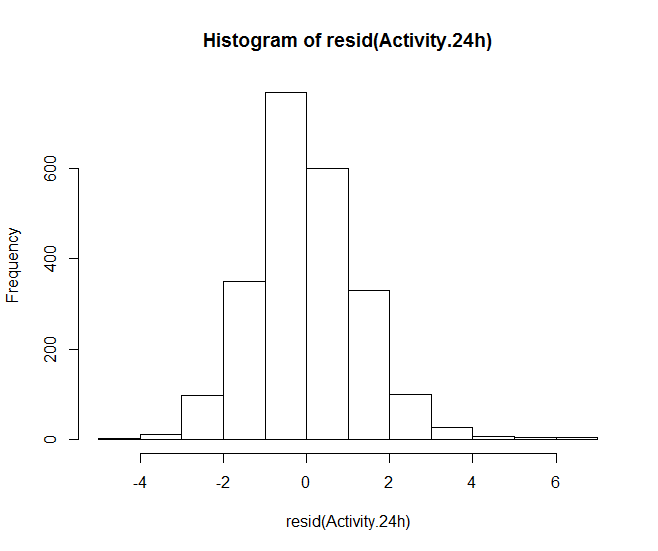 | 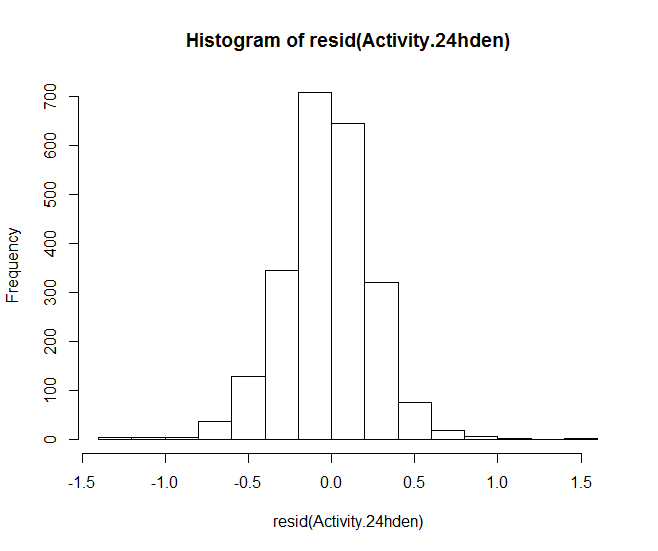 |

| Night-time range (denning and non-denning) | Daytime range (denning and nondenning) |
| --- | --- |
| 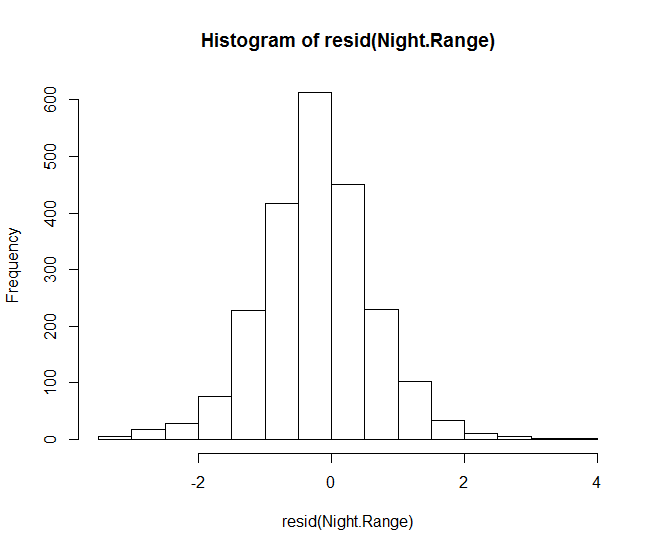 | 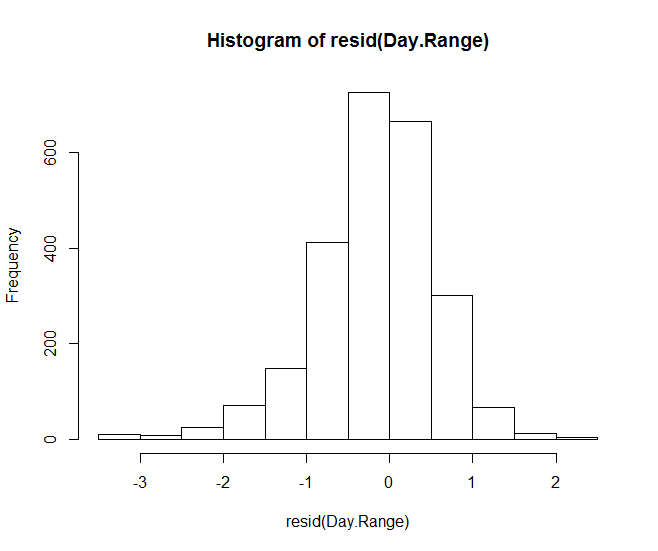 |
| Day-time range (denning) | Night-time range (denning) |
| 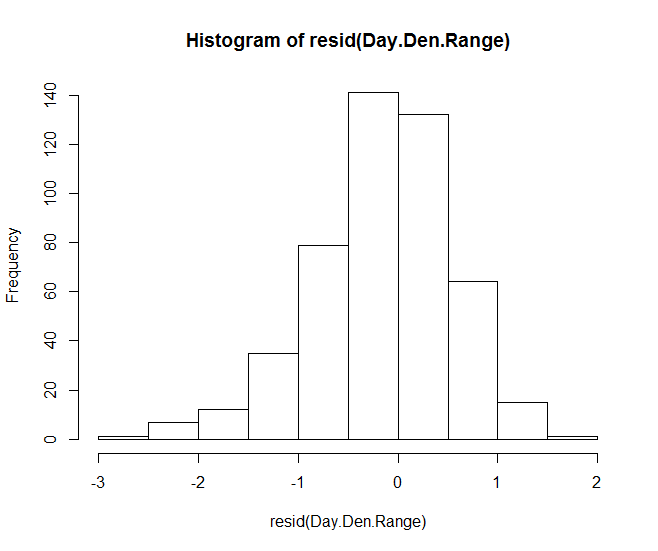 | 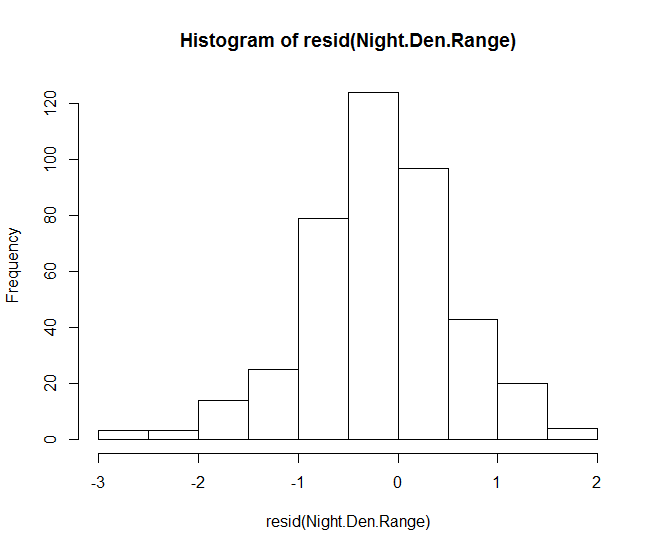 |
| 24 hour range (denning and non-denning) | 24 hour range (denning) |
| 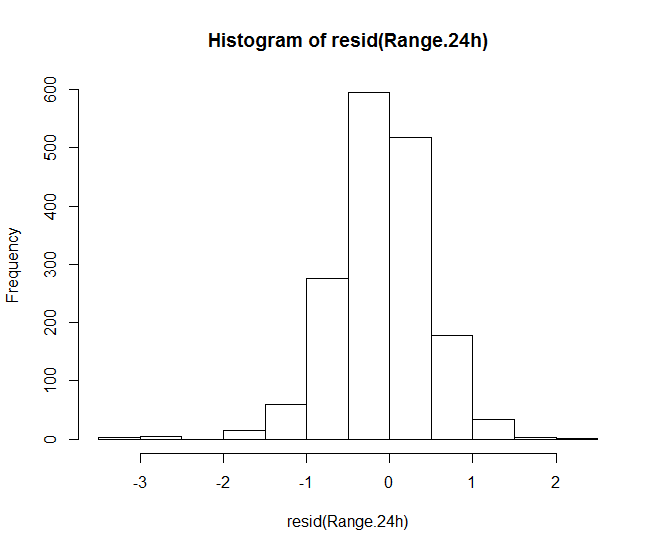 | 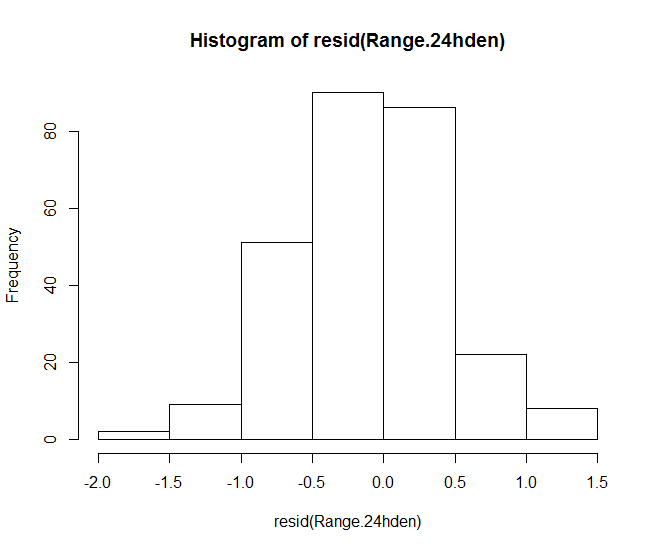 |

**Online Resource 7**: Q-Q plots of model residuals

| Night-time activity (denning and non-denning) | Daytime activity (denning and nondenning) |
| --- | --- |
| 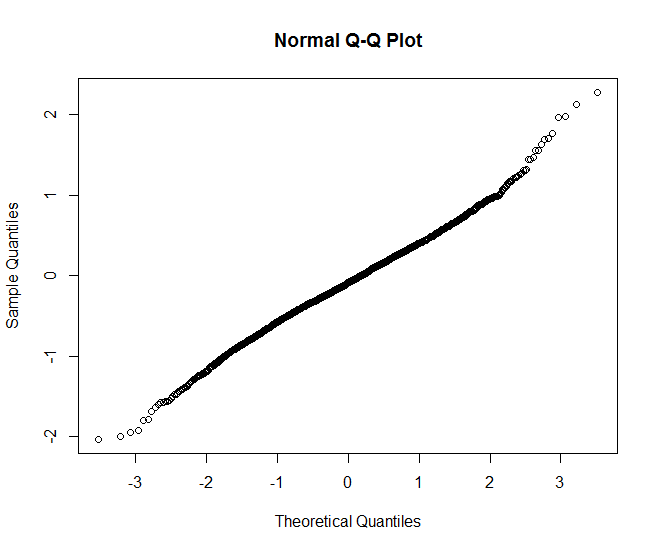 | 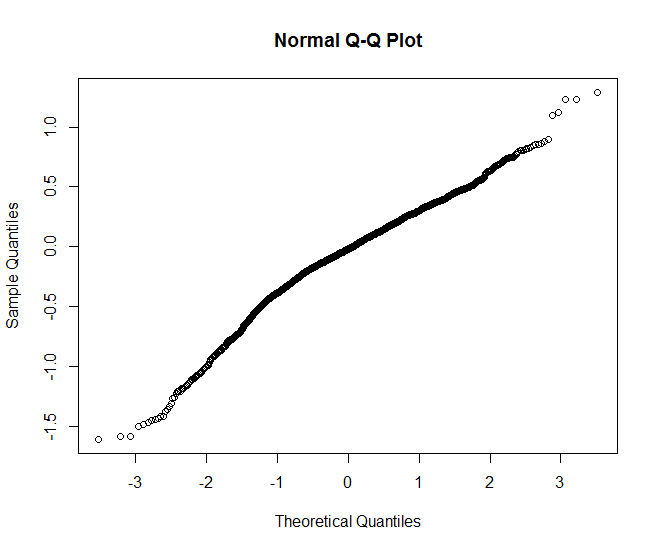 |
| Day-time activity (denning) | Night-time activity (denning) |
| 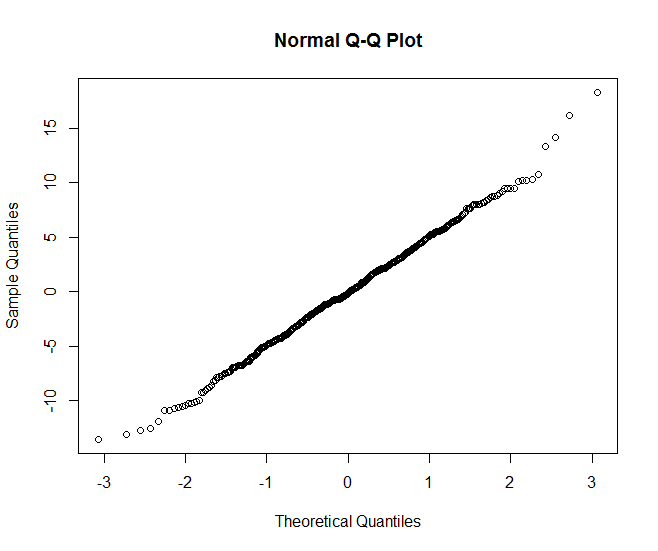 | 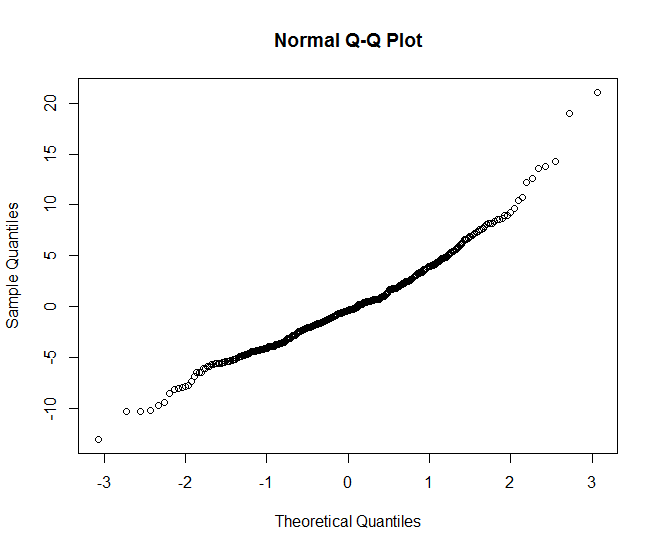 |
| 24 hour activity (denning and non-denning) | 24 hour activity (denning) |
| 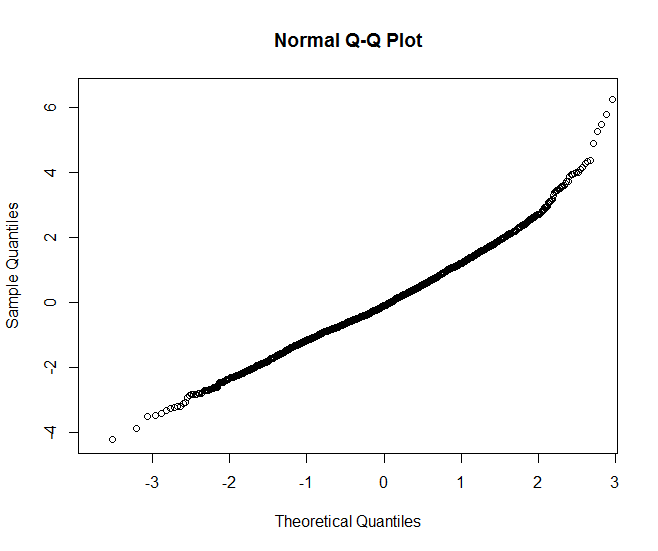 | 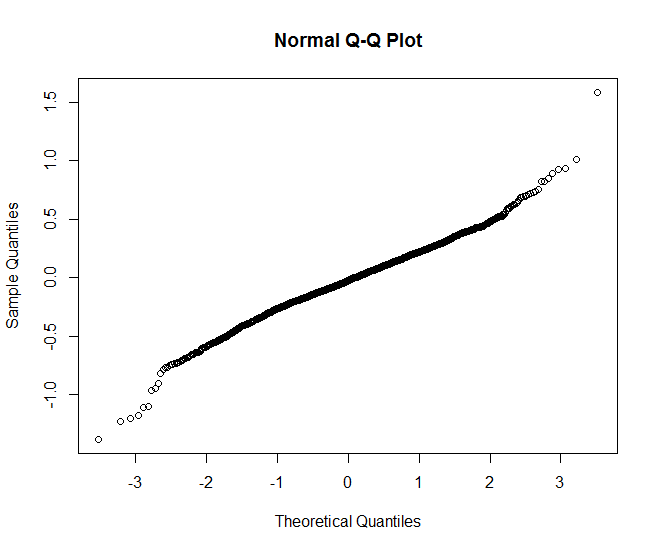 |

| Night-time range (denning and non-denning) | Daytime range (denning and nondenning) |
| --- | --- |
| 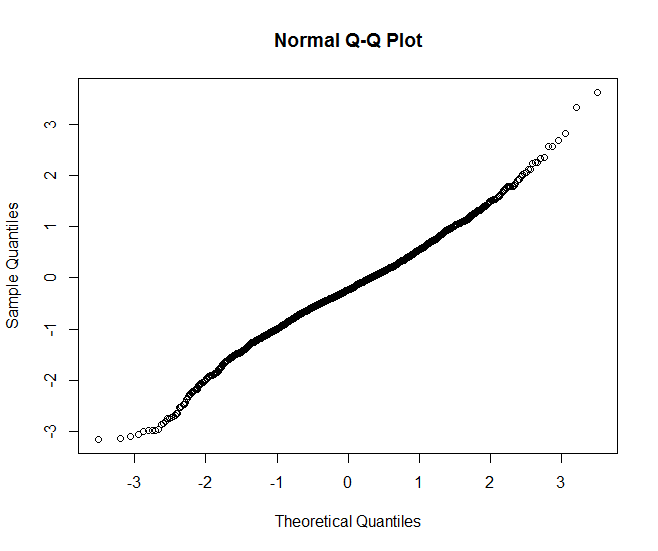 | 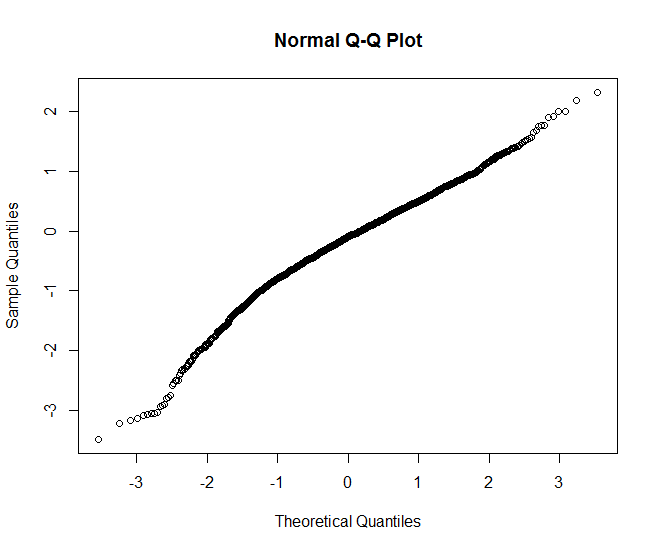 |
| Day-time range (denning) | Night-time range (denning) |
| 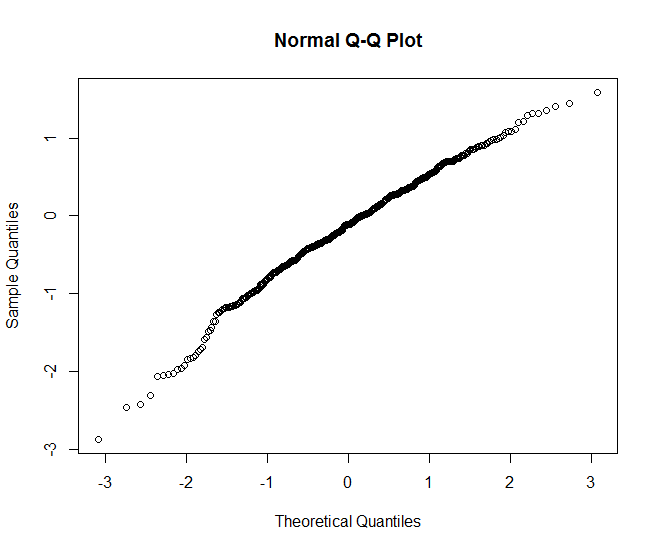 | 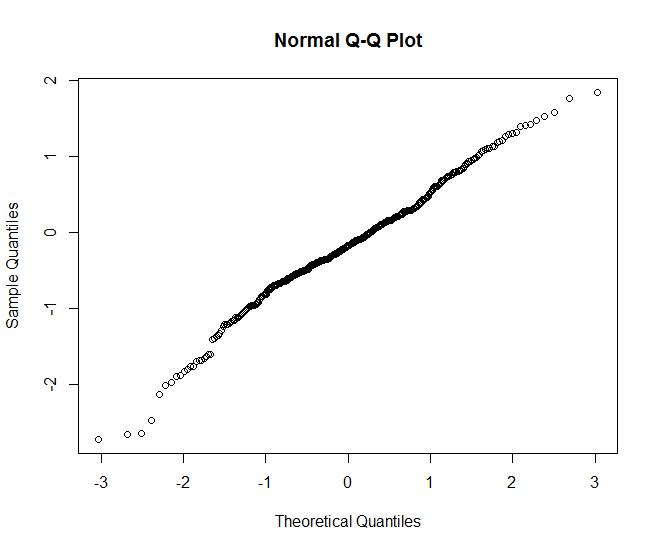 |
| 24 hour range (denning and non-denning) | 24 hour range (denning) |
| 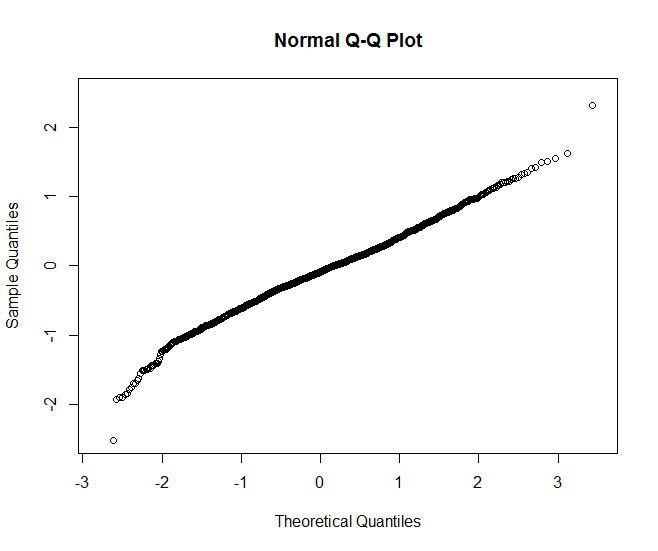 | 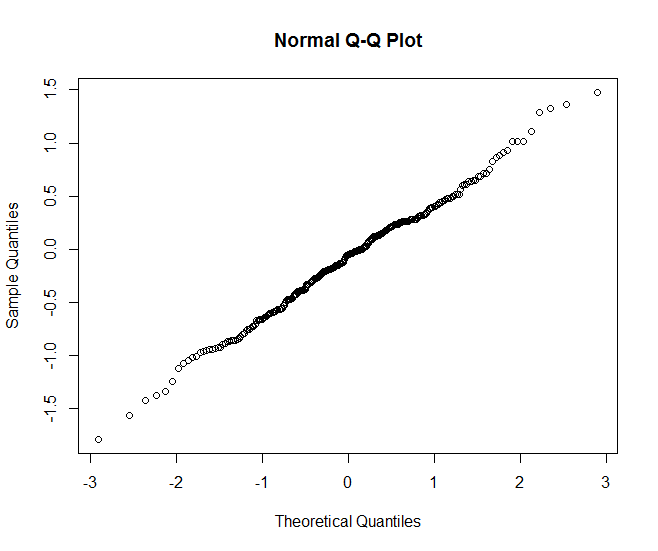 |

| Table OR8.1: list of models for **daylight hours** where delta < 5. | | | | | | |
| --- | --- | --- | --- | --- | --- | --- |
| 1= Maximum temperature | | 2= Rainfall | | | 3=Denning (Yes) | |
| 4=Maximum temperature*Rainfall | | 5=Maximum temperature*Denning (Yes) | | | 6=Days at den | |
| 7=Pup age | | 8=Days at den*Maximum temperature | | |  | |
| **Dependent variable** | **Time period** | | **Independent variables** | ***AICc*** | | ***Delta*** |
| ***Activity*** | All | | 1 2 3 4 | 14709.12 | | 0.00 |
|  |  |  | 1 2 3 4 5 | 14711.14 | | 2.01 |
|  |  |  | 1 2 3 | 14713.71 | | 4.58 |
|  | Denning | | 1 2 4 6 7 | 3281.10 | | 0.00 |
|  |  |  | 1 2 6 7 | 3281.92 | | 0.82 |
|  |  |  | 1 2 4 6 7 8 | 3283.09 | | 1.99 |
|  |  |  | 1 6 | 3283.82 | | 2.73 |
|  |  |  | 1 2 6 7 8 | 3283.83 | | 2.74 |
|  |  |  | 1 2 6 | 3285.14 | | 4.04 |
| ***Distance travelled*** | All | | 1 2 3 | 9679.18 | | 0.00 |
|  |  |  | 1 2 3 5 | 9680.09 | | 0.91 |
|  |  |  | 1 2 3 4 | 9680.12 | | 0.93 |
|  |  |  | 1 2 3 4 5 | 9680.87 | | 1.69 |
|  |  |  | 1 3 | 9683.28 | | 4.10 |
|  | Denning | | 1 6 7 | 1592.40 | | 0.00 |
|  |  |  | 1 6 | 1592.82 | | 0.43 |
|  |  |  | 6 7 | 1594.30 | | 1.90 |
|  |  |  | 1 2 6 | 1594.35 | | 1.96 |
|  |  |  | 1 2 6 7 | 1594.44 | | 2.04 |
|  |  |  | 1 2 4 6 7 | 1595.08 | | 2.68 |
|  |  |  | 6 | 1595.61 | | 3.21 |
|  |  |  | 1 2 6 7 8 | 1595.97 | | 3.58 |
|  |  |  | 2 6 7 | 1595.99 | | 3.60 |
|  |  |  | 1 2 4 6 7 8 | 1596.64 | | 4.24 |

**Online Resource 8**: Tables of top models

| Table OR8.2: list of models for **night-time hours** where delta < 5. | | | | | | |
| --- | --- | --- | --- | --- | --- | --- |
| 1= Maximum temperature | | 2= Rainfall | | | 3=Denning (Yes) | |
| 4=Maximum temperature*Rainfall | | 5=Maximum temperature*Denning (Yes) | | | 6=Days at den | |
| 7=Pup age | | 8=Days at den*Maximum temperature | | | 9=Moonlight | |
| **Dependent variable** | **Time period** | | **Independent variables** | ***AICc*** | | ***Delta*** |
| ***Activity*** | All | | 1 2 3 4 5 9 | 13401.64 | | 0.00 |
|  |  |  | 1 2 3 4 9 | 13403.49 | | 1.85 |
|  |  |  | 1 2 3 5 9 | 13404.91 | | 3.27 |
|  | Denning | | 9 | 2571.20 | | 0.00 |
|  |  |  | 1 9 | 2571.56 | | 0.36 |
|  |  |  | 6 9 | 2573.24 | | 2.04 |
|  |  |  | 1 2 9 | 2573.62 | | 2.42 |
|  |  |  | 1 2 5 6 9 | 2574.63 | | 3.43 |
|  |  |  | 1 6 7 9 | 2575.01 | | 3.80 |
|  |  |  | 1 2 7 9 | 2575.05 | | 3.85 |
|  |  |  | 1 2 6 9 | 2575.68 | | 4.48 |
| ***Distance travelled*** | All | | 1 2 3 5 9 | 10733.09 | | 0.00 |
|  |  |  | 1 3 9 | 10733.51 | | 0.42 |
|  |  |  | 1 2 3 4 5 9 | 10734.28 | | 1.20 |
|  |  |  | 1 2 3 9 | 10735.28 | | 2.20 |
|  |  |  | 1 2 3 4 9 | 10736.23 | | 3.14 |
|  | Denning | | 6 | 1555.51 | | 0.00 |
|  |  |  | 1 | 1556.16 | | 0.65 |
|  |  |  | 9 | 1556.29 | | 0.79 |
|  |  |  | 7 | 1556.30 | | 0.79 |
|  |  |  | 2 | 1556.30 | | 0.80 |
|  |  |  | 1 6 | 1556.46 | | 1.96 |
|  |  |  | 6 9 | 1556.55 | | 2.05 |
|  |  |  | 1 9 | 1556.19 | | 2.68 |
|  |  |  | 1 2 9 | 1556.25 | | 4.75 |
|  |  |  | 1 2 | 1556.29 | | 4.78 |

| Table OR8.3: list of models for the **24 hour period** where delta < 5. | | | | | | |
| --- | --- | --- | --- | --- | --- | --- |
| 1= Maximum temperature | | 2= Rainfall | | | 3=Denning (Yes) | |
| 4=Maximum temperature*Rainfall | | 5=Maximum temperature*Denning (Yes) | | | 6=Days at den | |
| 7=Pup age | | 8=Days at den*Maximum temperature | | | 9=Moonlight | |
| **Dependent variable** | **Time period** | | **Independent variables** | ***AICc*** | | ***Delta*** |
| ***Activity*** | All | | 1 2 3 5 9 | 12312.72 | | 0.00 |
|  |  |  | 1 2 3 4 5 9 | 12312.97 | | 0.25 |
|  |  |  | 1 2 3 9 | 12314.25 | | 1.52 |
|  |  |  | 1 2 3 4 9 | 12314.82 | | 2.09 |
|  |  |  | 2 3 5 9 | 12316.82 | | 4.10 |
|  | Denning | | 1 2 6 7 8 9 | 2747.22 | | 0.00 |
|  |  |  | 1 2 6 9 | 2747.64 | | 0.42 |
|  |  |  | 2 6 9 | 2747.81 | | 0.59 |
|  |  |  | 1 2 4 6 7 8 9 | 2748.70 | | 1.48 |
|  |  |  | 1 2 6 7 9 | 2749.07 | | 1.85 |
|  |  |  | 2 6 7 9 | 2749.10 | | 1.88 |
|  |  |  | 1 2 6 | 2749.29 | | 2.08 |
|  |  |  | 1 2 4 6 7 9 | 2750.27 | | 3.05 |
|  |  |  | 1 6 9 | 2750.94 | | 3.72 |
|  |  |  | 1 2 6 7 | 2750.98 | | 3.77 |
|  |  |  | 1 6 | 2751.04 | | 3.82 |
|  |  |  | 1 2 7 9 | 2751.84 | | 4.63 |
| ***Distance travelled*** | All | | 1 2 3 5 9 | 9416.02 | | 0.00 |
|  |  |  | 3 9 | 9416.91 | | 0.89 |
|  |  |  | 9 | 9417.38 | | 1.36 |
|  |  |  | 1 2 3 4 5 9 | 9417.82 | | 1.80 |
|  |  |  | 2 3 9 | 9418.19 | | 2.16 |
|  |  |  | 3 9 | 9418.55 | | 2.53 |
|  |  |  | 3 | 9419.01 | | 2.99 |
|  |  |  | 1 9 | 9419.34 | | 3.31 |
|  |  |  | 1 2 3 9 | 9419.91 | | 3.89 |
|  |  |  | 1 3 | 9420.50 | | 4.48 |
|  |  |  | 1 2 9 | 9420.65 | | 4.62 |
|  |  |  | 2 | 9420.92 | | 4.89 |
|  | Denning | | 1 6 | 1308.19 | | 0.00 |
|  |  |  | 6 | 1308.25 | | 0.06 |
|  |  |  | 1 | 1308.33 | | 0.13 |
|  |  |  | 6 7 | 1308.52 | | 0.32 |
|  |  |  | 1 2 6 | 1310.20 | | 2.01 |
|  |  |  | 7 | 1310.25 | | 2.06 |
|  |  |  | 1 9 | 1310.29 | | 2.09 |
|  |  |  | 1 2 | 1310.33 | | 2.13 |
|  |  |  | 1 6 7 9 | 1310.43 | | 2.24 |
|  |  |  | 2 | 1310.67 | | 2.48 |
|  |  |  | 1 2 6 7 | 1310.74 | | 2.55 |
|  |  |  | 9 | 1311.03 | | 2.83 |
|  |  |  | 1 2 6 9 | 1312.12 | | 3.93 |
|  |  |  | 1 2 9 | 1312.29 | | 4.10 |
|  |  |  | 1 2 6 7 9 | 1312.56 | | 4.36 |
|  |  |  | 2 6 9 | 1312.57 | | 3.37 |

**Online Resource 9**: Table of correlations between explanatory variables

| **Variable 1** | **Variable 2** | **Correlation** |
| --- | --- | --- |
| Days at den | Pup age | 0.23 |
| Days at den | Maximum temperature | -0.12 |
| Days at den | Rainfall | -0.06 |
| Days at den | Moonlight | 0.02 |
| Pup age | Maximum temperature | 0.04 |
| Pup age | Rainfall | -0.16 |
| Pup age | Moonlight | -0.13 |
| Maximum temperature | Rainfall | -0.14 |
| Maximum temperature | Moonlight | -0.07 |
| Rainfall | Moonlight | 0.02 |

**28**

**29**

**31**

**32**

**29**

**29**

**30**

**31**

**29**

**30**

**32**

**33**

**31**

**31**

**33**

**33**

**January**

**February**

**April**

**March**

**12**

**months**

**Worldclim current temperatures**

**(mean daily maximum)**

**Mean**

**29**

**30**

**31**

**32**

**Mean monthly current temperature**

**29**

**30**

**32**

**33**

**33**

**32**

**35**

**34**

**31**

**31**

**36**

**35**

**32**

**32**

**37**

**36**

**January**

**February**

**April**

**March**

**12**

**months**

**Worldclim future predictions**

**(mean daily maximum)**

**Mean**

**32**

**32**

**35**

**34**

**Mean monthly future temperature**

**32**

**32**

**35**

**34**

**Mean monthly future temperature**

**29**

**30**

**31**

**32**

**Mean monthly current temperature**

**+3**

**+2**

**+4**

**+2**

**Mean monthly difference**

**Mean**

**2.75**

**Mean temperature change**

**2.75**

**Mean temperature increase**

**27.6**

**Mean weather station temperature**

**30.35**

**Temperature for predictions**

**Online Resource 10:** Diagrams showing the calculations performed on the Worldclim temperature and rainfall data to obtain the values that were entered to obtain future projections of activity and distances travelled. Numbers used are examples and do not reflect the data.

**50**

**40**

**35**

**45**

**50**

**55**

**40**

**50**

**55**

**55**

**40**

**50**

**55**

**60**

**45**

**55**

**January**

**February**

**April**

**March**

**12**

**months**

**Worldclim current rainfall**

**(monthly total)**

**Mean**

**50**

**55**

**45**

**45**

**Mean current total monthly rainfall**

**45**

**35**

**25**

**40**

**45**

**50**

**35**

**45**

**50**

**45**

**45**

**45**

**50**

**50**

**35**

**45**

**January**

**February**

**April**

**March**

**12**

**months**

**Worldclim future predictions**

**(monthly total)**

**Mean**

**45**

**45**

**35**

**40**

**Mean future total monthly rainfall**

**45**

**45**

**35**

**40**

**Mean future total monthly rainfall**

**50**

**55**

**45**

**45**

**Mean current total monthly rainfall**

**-5**

**-10**

**-10**

**-5**

**Mean monthly difference**

**Mean**

**-7.5**

**Mean monthly total rainfall change**

**-7.5/30**

**Mean daily rainfall change**

**1.6**

**Mean daily weather station rainfall**

**1.35**

**Rainfall for predictions**

**Online Resource 11**: Predicted minimum, mean and maximum differences in daily total precipitation and mean maximum temperature across the study site in the best (RCP 2.6) and worst case (RCP 8.5) HADGEM2 climate scenarios from Worldclim 1.4 between 2010 and 2070.

|  |  | Minimum | Mean | Maximum |
| --- | --- | --- | --- | --- |
| Temperature (°C) | Best case | -0.6 | 1.6 | 3.3 |
|  | Worst case | 1.8 | 3.9 | 5.9 |
| Rainfall (mm) | Best case | -0.6 | -0.2 | 0.2 |
|  | Worst case | -0.7 | -0.5 | 0.1 |

**Online Resource 12**: Study area maps showing projected change in mean maximum temperature (°C) and average total daily precipitation (mm) between 2010 and 2070 for the best (Representative Concentration Pathway 2.6) and worst (Representative Concentration Pathway 8.5) case IPCC scenarios: a) predicted change in temperature under RCP 2.6; b) predicted change in temperature under RCP 8.5; c) predicted change in precipitation under RCP 2.6; d) predicted change in precipitation under RCP 8.5. RCP 2.6 indicates the best case IPCC scenario, and RCP 8.5 the worst case. Predicted future temperatures are from the HADGEM2 climate model.


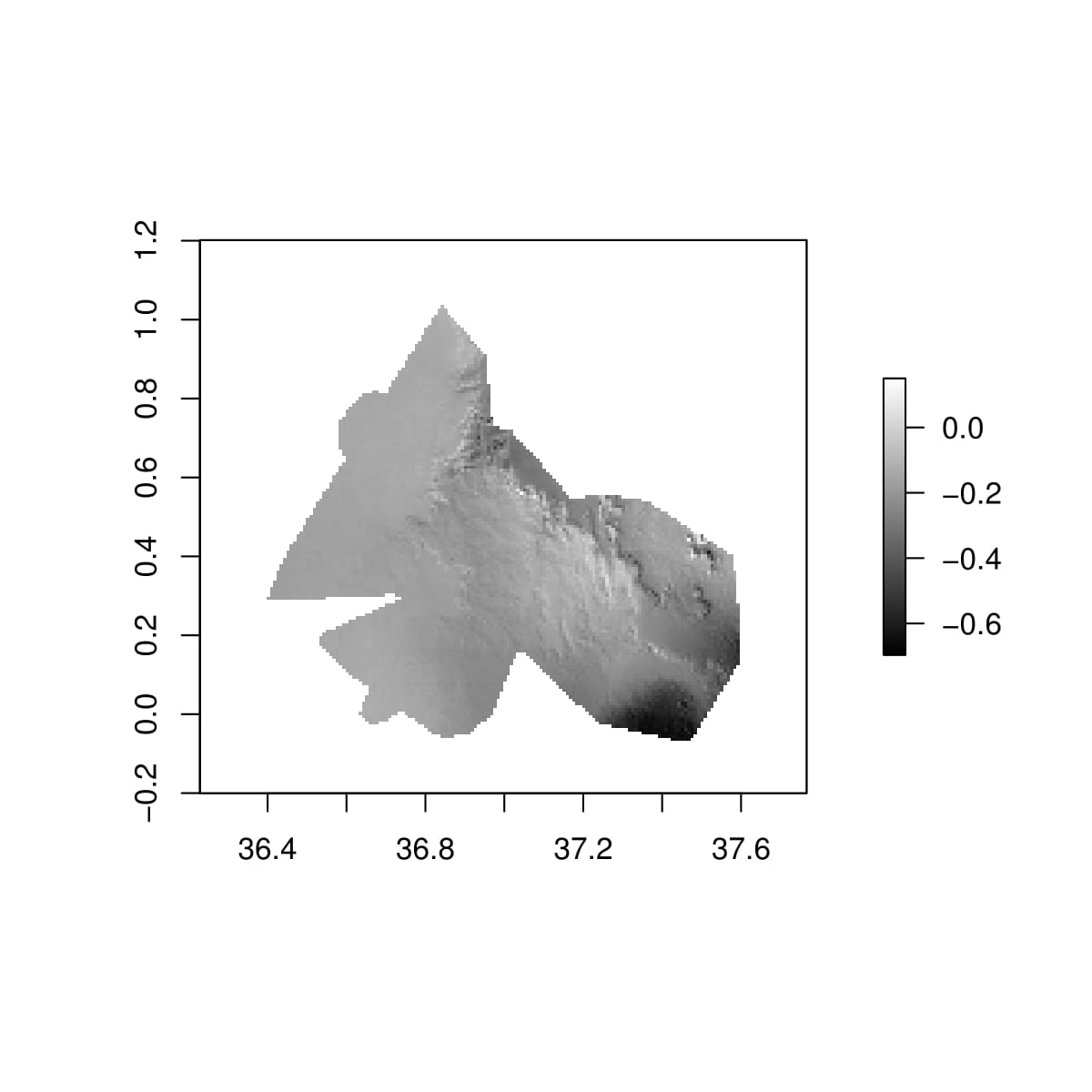

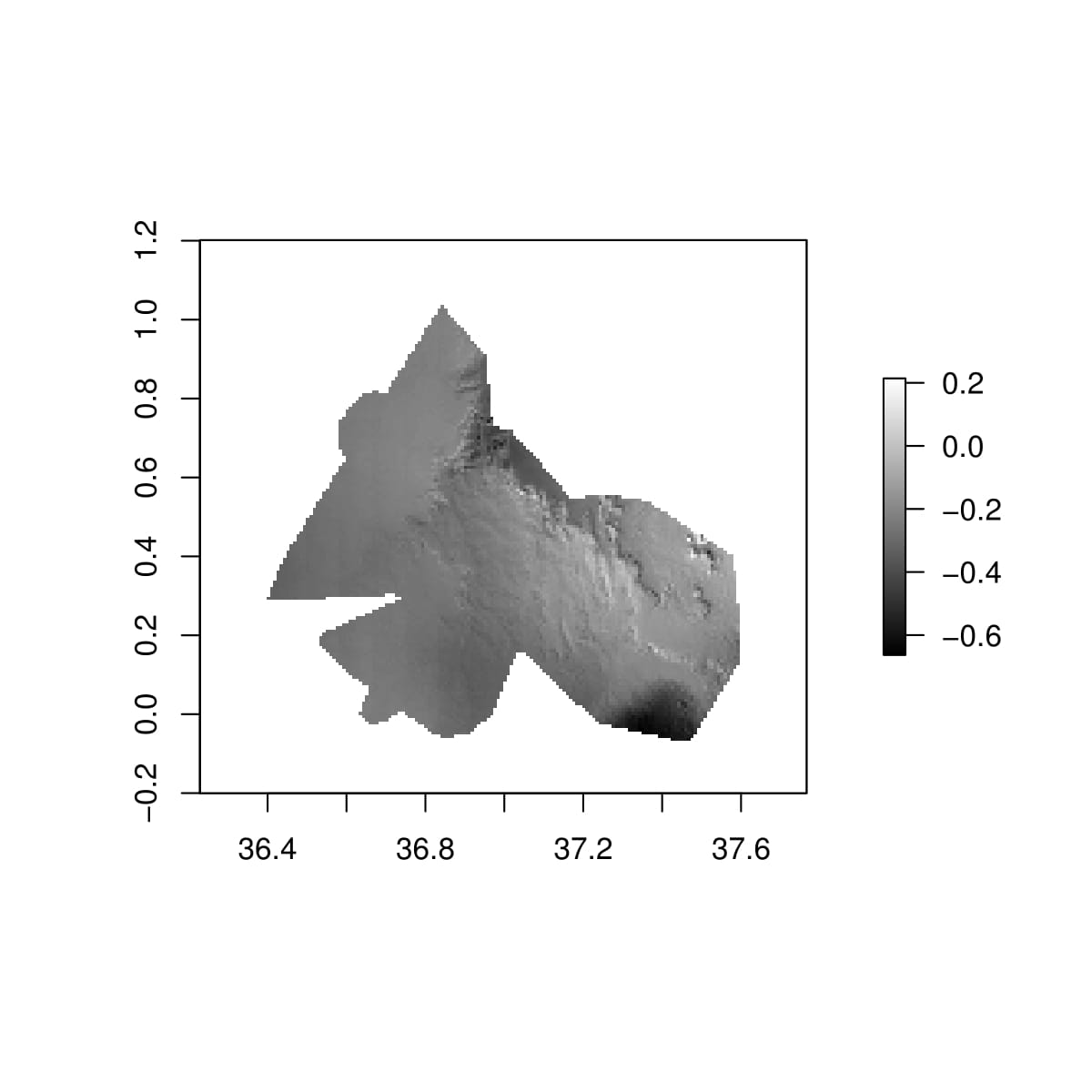

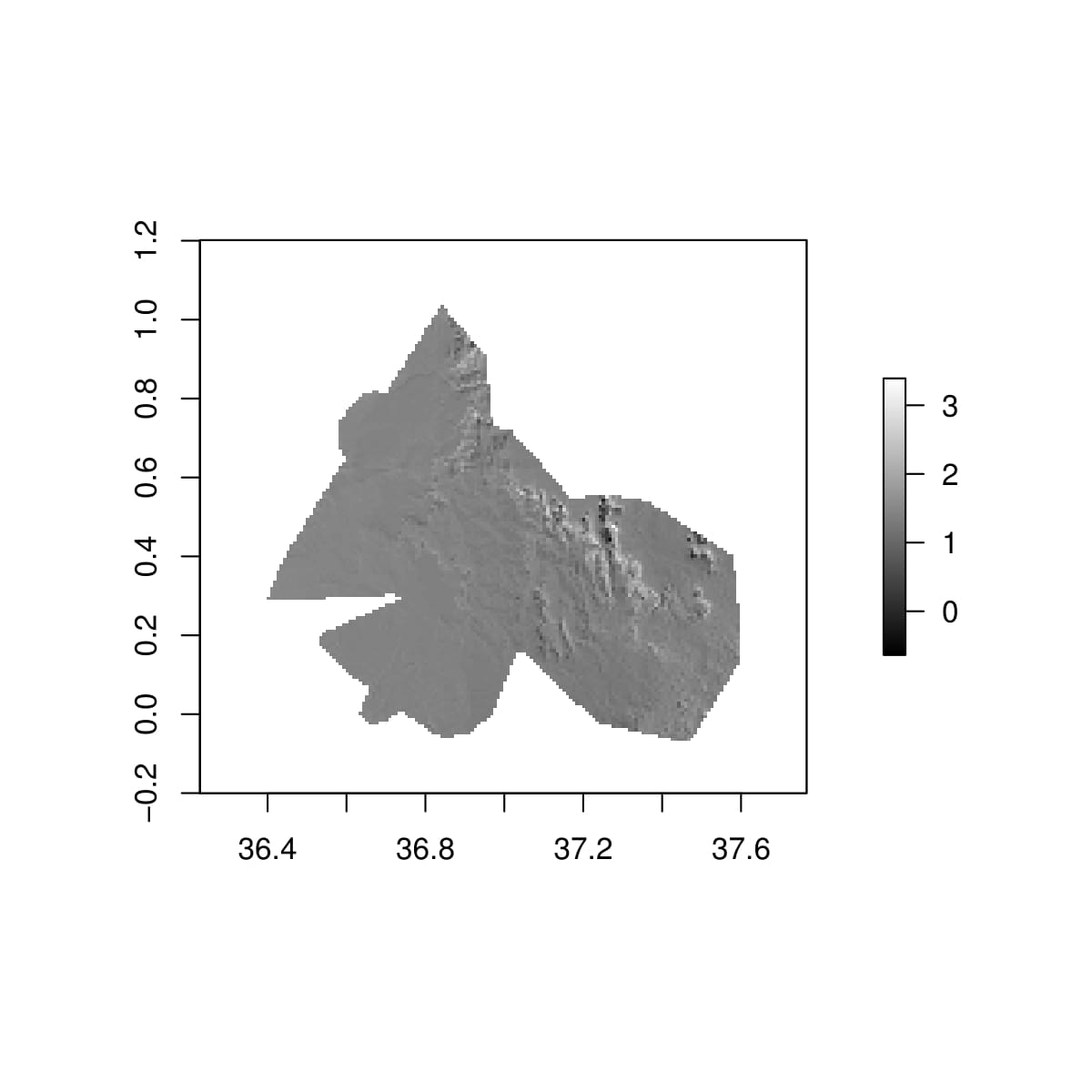

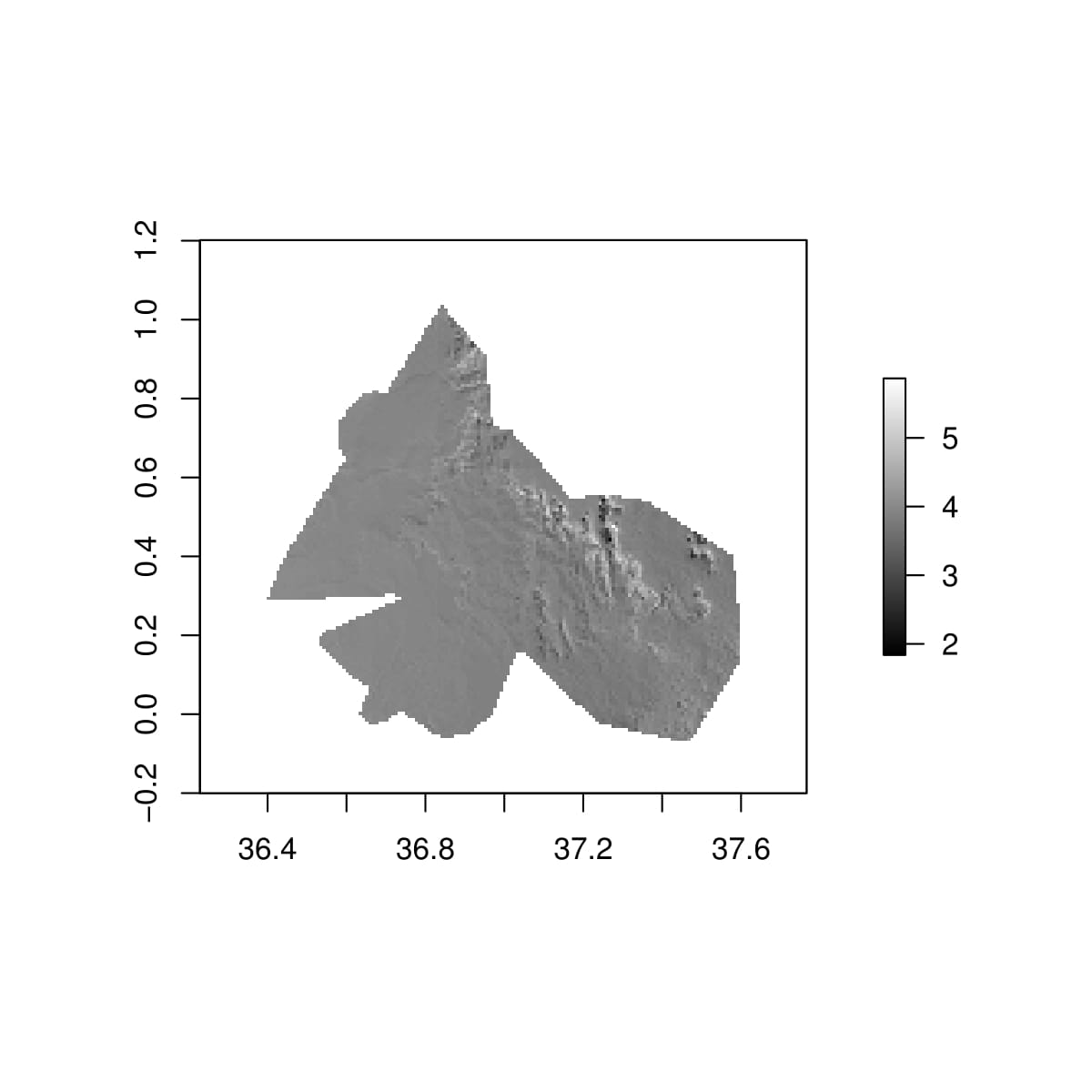


a)

b)

c)

d)

Latitude

Longitude

Latitude

Longitude

Best case

Worst case

°C

°C

mm

mm

**Online Resource 13**: Histogram of moonlight showing a high proportion of nights have low levels of moonlight


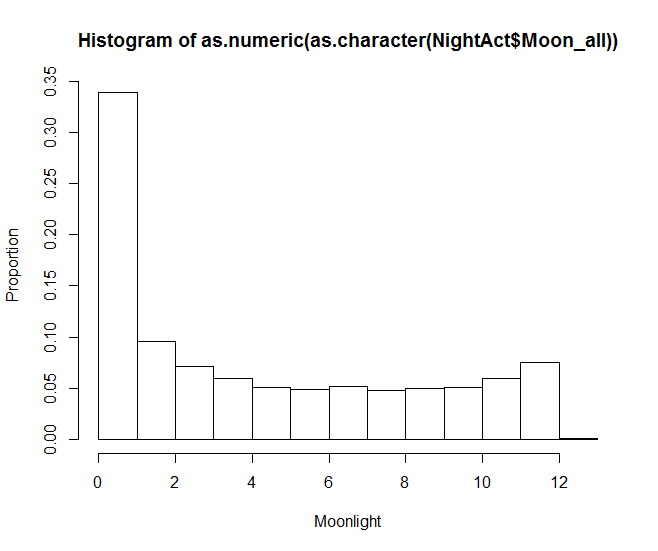


**Online Resource 13:** Scatter plots of raw data and model predictions.

14.1: Estimated mean activity during (a) outside the denning period and (b) inside the denning period. Points represent the raw data. Unbroken lines show activity levels predicted by the models at mean rainfall and moonlight levels. Dashed lines represent confidence intervals.

14.2: Overall estimated mean nocturnal activity at high and low moonlight levels. Points represent the raw data, with low moonlight categorised as moonlight values between 0 and 1 and high moonlight categorised as values between 11 and 12. Unbroken lines show activity levels predicted by the models at mean rainfall levels. High moonlight is the estimated activity levels at moonlight value 12 and low moonlight is moonlight value 0. Dashed lines represent confidence intervals.

14.3: Estimated mean activity across a 24 hour period from dawn to dawn. Points represent the raw data. Unbroken lines show activity levels predicted by the models at mean rainfall and moonlight levels. Dashed lines represent confidence intervals.


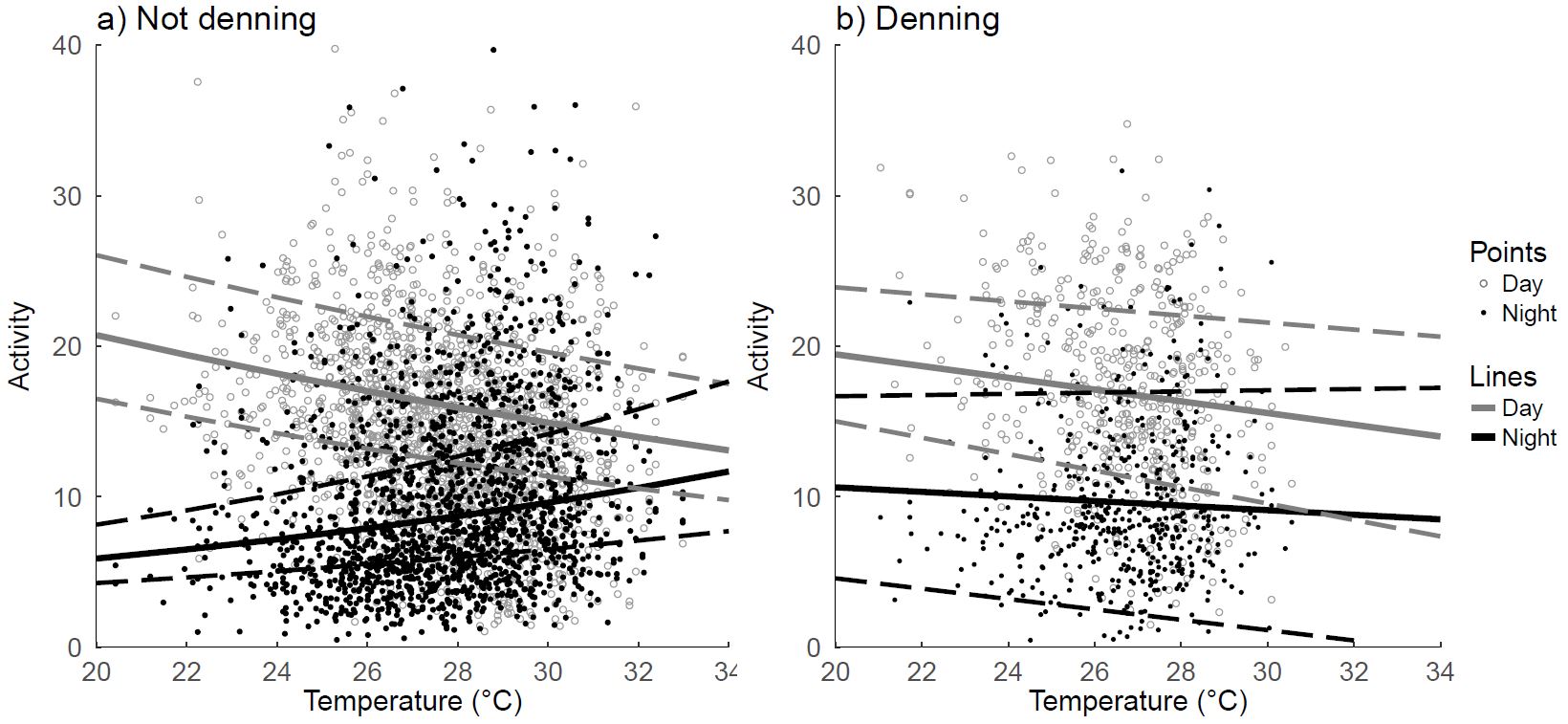


**14.1**


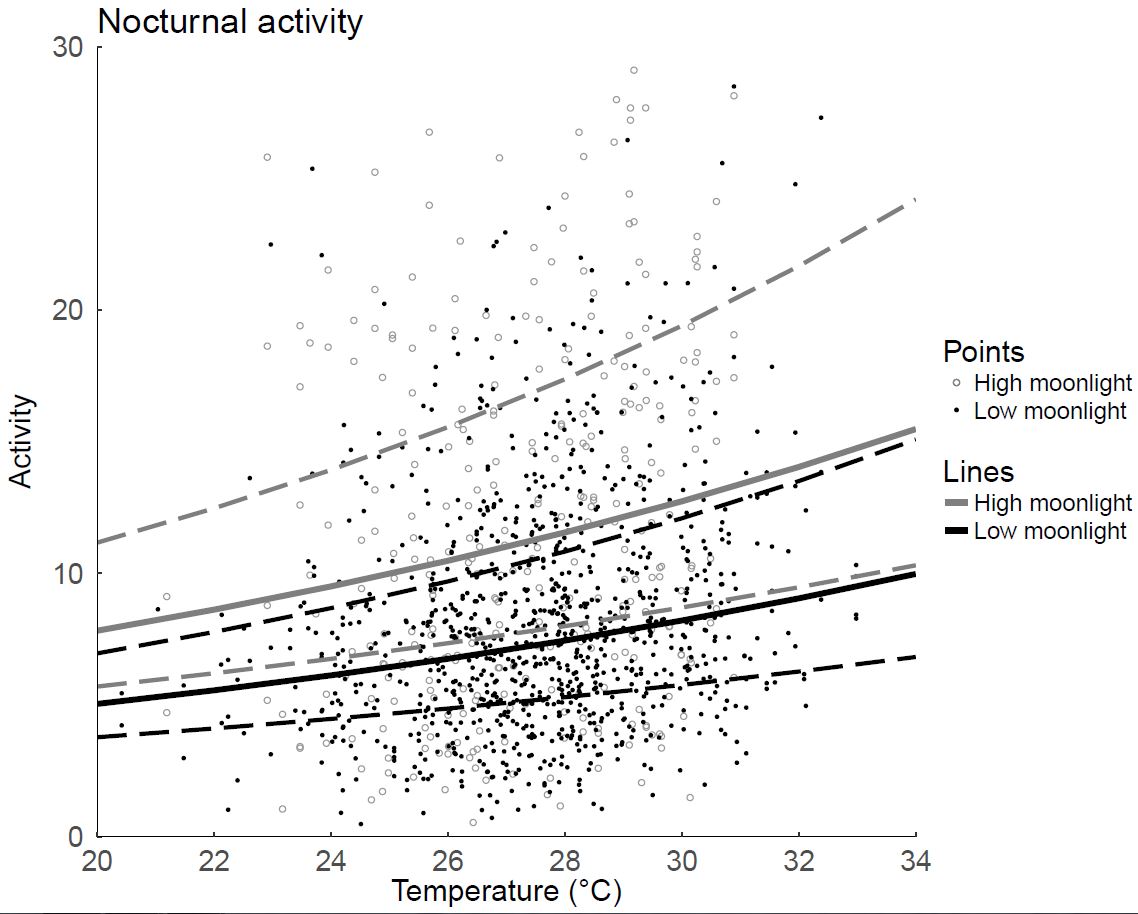


**14.2**


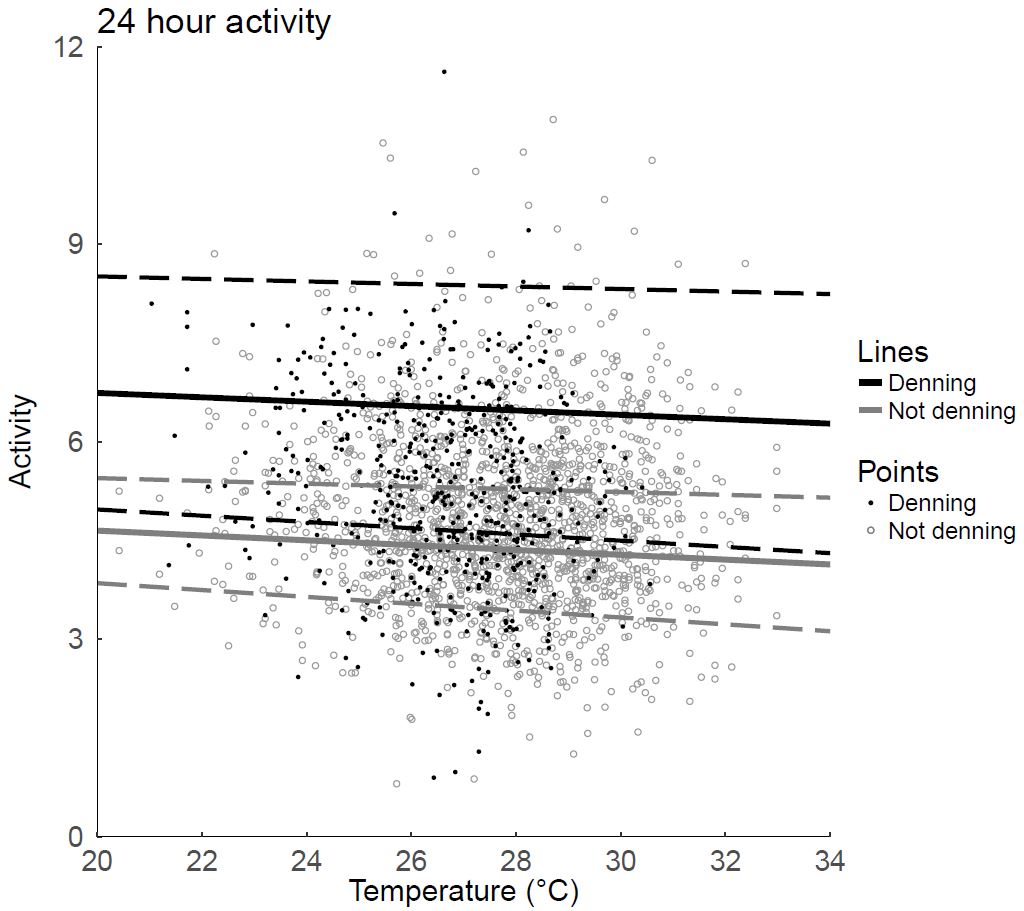


**14.2**
